# Microbiological quality and antimicrobial resistance of *Pseudomonas aeruginosa* in drinking water systems of the old terminal of Jorge Chávez International Airport: potential role as environmental reservoir

**DOI:** 10.17843/rpmesp.2026.432.15160

**Published:** 2026-06-30

**Authors:** Leonardo Enrique Sulca-Galarza, Rigoberto Cilis Robles-Camarena, Tito Libio Sánchez-Rojas, Aldo Franco Rodríguez-Gonzales

**Affiliations:** 1 Dirección de Sanidad Aérea Internacional, Callao, Peru.; 2 Facultad de Ciencias Biológicas, Universidad Nacional Mayor de San Marcos, Lima, Peru.; 3 Dirección Ejecutiva de Sanidades Internacionales, Callao, Peru.; 4 Laboratorio de Biotecnología Ambiental y Bioprocesos industriales, Facultad de Ciencias Biológicas, Universidad Nacional Mayor de San Marcos, Lima, Peru.

**Keywords:** Pseudomonas aeruginosa, Drinking Water, Drug Resistance, Microbial, Airports, Health Surveillance, Water Quality

## Abstract

**Objective:**

To determine the microbiological quality of water for human consumption at an international airport in Peru according to national regulations, to evaluate the phenotypic antimicrobial resistance patterns of Pseudomonas aeruginosa isolated from water systems, and to explore its possible role as an environmental reservoir of resistant bacteria at an international entry point.

**Materials and Methods:**

Descriptive cross-sectional study conducted in 2024. A total of 145 water samples from airport terminal taps, restaurants, supplier companies, wells, and aircraft were analyzed. Microbiological indicators were evaluated according to current national regulations, and the antimicrobial susceptibility of 67 P. aeruginosa isolates was determined by disk diffusion. MDR, XDR, and PDR classifications were established according to international criteria.

**Results:**

50.3% (73/145) of the samples did not meet the microbiological standards established by the regulations. P. aeruginosa was detected in 55.8% (81/145) of the samples. Of the 67 isolates analyzed, 38.8% were classified as MDR and 11.9% as XDR; no PDR isolates were identified. Concentrations exceeding 16 MPN/100 mL were observed at multiple points in the system, including aircraft.

**Conclusions:**

The detection of resistant P. aeruginosa in airport water systems suggests sanitary vulnerabilities and a possible role as an environmental reservoir of resistant bacteria. It is recommended to strengthen microbiological surveillance and incorporate antimicrobial resistance assessment into water quality monitoring programs at international entry points.

## INTRODUCTION

Jorge Chavez International Airport in Callao (AIC), whose operations were moved to a new passenger terminal, constitutes Peru's main international air entry point and concentrates a high flow of passengers, aircraft, and associated operational activities ^1^. These characteristics make it an environment of interest for health surveillance and for the evaluation of the microbiological quality of water intended for human consumption in complex distribution and supply systems.

In this regard, the risk to public health is determined by the severity and development of diseases associated with pathogenic agents, their infectivity, and the exposed population ^2^. However, in vulnerable populations such as infants, young children, immunologically weakened or immunocompromised individuals, and the elderly ^3^, their main mode of transmission is the consumption of contaminated water; but there are other routes such as person-to-person contact, food intake, droplets, and aerosols ^2^. Therefore, to guarantee the quality of water for human consumption and protect public health in airports, it is essential to implement safety frameworks and comprehensive supply security plans, in order to prevent outbreaks of waterborne diseases that could affect numerous travelers ^2^.

Bacterial resistance is considered one of the threats to global public health, with millions of attributable deaths annually and increasing projections towards 2050 ^4^. The distribution, prevalence, and diversity of antimicrobial-resistant bacteria show marked differences among different regions of the world, due to the influence of health factors, socioeconomic conditions, genetics, and environmental variables ^4^^,^^5^. Furthermore, this heterogeneity intensifies in the context of globalization, since the increase in international travel and cross-border trade facilitates the exchange or horizontal transfer of genes and the global dissemination of resistant bacteria ^6^. It is probable that passengers infected with pathogens eliminate them in their feces and bodily fluids (urine, saliva, sputum, etc.) when using airport and aircraft sanitary facilities ^7^.

According to DS 031-2010-SA ^8^, the main microbiological indicator of drinking water quality are coliforms, especially thermotolerant coliforms and *Escherichia coli*, as evidence of recent fecal contamination ^9^. The standard establishes limits of <1.1 MPN/100 mL or <1 CFU/100 mL; however, a single indicator does not guarantee safety against the diversity of potential pathogens. In this context, *Pseudomonas aeruginosa* is not considered in routine drinking water control, although it does appear as a microbiological criterion in bottled beverages ^10^ and in the current health regulations for collective use swimming pools ^11^, thus demonstrating a certain lack or bias in Peruvian regulations on drinking water quality, which excludes *Pseudomonas aeruginosa* from microbiological control, a Gram-negative bacillus known for its resistance to multiple antibiotics, an opportunistic pathogen that can contaminate water and represent a significant risk to human health, classified as high priority due to its carbapenem resistance ^12^. Its ability to survive in adverse environments and develop resistance to antimicrobial agents makes it a relevant indicator for evaluating the microbiological quality of water, in addition to its ability to inhibit coliforms ^13^. On the other hand, the absence of coliform bacteria in cistern and tank water samples does not mean the absence of microbiological risk, and *Pseudomonas aeruginosa* can be found due to its versatile metabolism, resistance, and as an opportunistic pathogen ^14^.

In 2019, *Pseudomonas aeruginosa* generated approximately 84,600 deaths attributable to antimicrobial resistance to one or more antibiotics ^4^. When analyzing pathogen-drug combinations, the highest mortality burden was observed in carbapenem resistance (≈38,100 deaths), followed by fluoroquinolones (≈18,300 deaths) and antibiotics with antipseudomonal activity (≈10,300 deaths). Deaths attributable to resistance to third-generation cephalosporins (≈10,400), fourth-generation cephalosporins (≈4,370), and aminoglycosides (≈3,010) were also recorded. These findings show that, although *Pseudomonas aeruginosa* maintains a high burden of resistance-associated mortality, the greatest clinical impact is concentrated in the loss of efficacy of carbapenems, considered last-line drugs in severe infections ^4^.

The present study evaluates the quality of water for human consumption at different points of the AIC, with emphasis on the detection and antimicrobial resistance of *Pseudomonas aeruginosa*, a bacterium considered by the World Health Organization (WHO) as a priority pathogen ^12^. In this context, it seeks to generate updated evidence on water contamination and the presence of resistant strains, in order to identify health risks at an international entry point.

**KEY MESSAGES**

Motivation for the study. There is limited evidence on the presence of Pseudomonas aeruginosa and its antimicrobial resistance in drinking wáter systems at airports in Latin America, despite their relevance as points of high international connectivity.

Main findings. A considerable percentage of samples was identified as not complying with the microbiological standards of national and international regulations, as well as a significant frequency of P. aeruginosa strains with antimicrobial resistance, including multidrug-resistant and extensively drug-resistant phenotypes.

Public health implications. The results suggest that airport water systems could act as potential environmental reservoirs for resistant bacteria, which supports the need to strengthen microbiological surveillance and update sanitary regulations.

## MATERIALS AND METHODS

### Design and study area

A descriptive, cross-sectional observational study was conducted at the AIC, located in the Constitutional Province of Callao. The AIC is considered one of the three most important airports in South America and is under the jurisdiction of the Regional Health Directorate (DIRESA) Callao. It operates 24 hours a day and provides services to national and international commercial airlines, with 24 international and 5 national airlines. In 2023, the airport registered approximately 21.2 million passengers, which represented a 14.1% increase compared to the previous year; it is expected that this figure will increase significantly with the entry into operation of the new airport terminal ^1^.

### Sampling points

The selection of sampling points was carried out under a health risk approach, prioritizing critical transfer and consumption points defined in the Guide to Hygiene and Sanitation in Air Transport and within the framework of the Water Safety Plan for airports; 16 sampling units were considered: 9 fixed points, 6 coded variable points, and an additional category corresponding to commercial aircraft. The fixed points included faucets in public access restrooms, restaurant faucets, the kitchen faucet of the International Air Health Directorate, and a water well for human consumption that supplies the airport terminal. The coded variable points corresponded to tanker trucks supplying water for aircraft, a water dispenser for human consumption, and a well associated with said system. Additionally, samples from commercial aircraft were included, which were considered a variable sampling unit because the aircraft evaluated varied during the study period.

Fixed points were evaluated monthly throughout the entire study period, generating repeated measurements at the same sampling points. Variable points were evaluated according to operational availability and supply system conditions. In the case of companies supplying water for aircraft, a quarterly rotating sampling was planned among the companies; however, due to operational limitations, it was not possible to strictly maintain said rotation. Nevertheless, 4 evaluations per company were carried out during the year, obtaining 2 samples per visit, reaching a total of 8 samples per company.

Regarding the aircraft, the samples were obtained from different units on each occasion, corresponding to 5 international and 8 national flights, therefore, these observations are considered independent from each other.

### Number of samples and sampling time

The total monthly samples collected were around 12, between January and December 2024, giving a total of 145 samples (13 samples were taken in December). These samples included both fixed points repeatedly evaluated and variable points non-systematically evaluated throughout the study period.

The collection, transport, and preservation of water samples was carried out by professional personnel and environmental health technicians of the International Air Sanitation Directorate, following the recommendations of the American Public Health Association, American Water Works Association, and Water Environment Federation (APHA, AWWA, and WEF) ^15^.

### Microbiological quality and methodology

Microbiological evaluations were performed on each individual collected sample, following standardized procedures for the evaluation of the microbiological quality of water for human consumption. The bacterial indicators recommended by regulation DS 031-2010-SA Regulation on the quality of potable water for human consumption ^8^ were used: total coliforms, thermotolerant coliforms, *Escherichia coli* and heterotrophic bacteria; additionally, *Pseudomonas aeruginosa* was included as a complementary indicator of sanitary risk according to the Guide to Hygiene and Sanitation in Air Transport and WHO Guidelines for Drinking-water Quality.

The methodology used for determining the microbiological quality of drinking water was the multiple-tube fermentation technique by most probable number (MPN/100 mL), in accordance with the standard methods of APHA, AWWA, WEF ^15^. The method's detection limit was <1.1 MPN/100 mL, while the upper limit was >23 MPN/100 mL. For *Pseudomonas aeruginosa*, the method by Ramírez Z. ^9^, Álvarez Guevara J. et al. ^16^ was modified, and confirmation criteria were followed according to ISO 16266:2006 ^17^. Its method's detection limit was <2.2 MPN/100 mL, while the upper limit was >16 MPN/100 mL.

For the quantification of heterotrophic bacteria, Plate Count agar by pouring was used according to method 9215 Heterotrophic plate count of the APHA AWWA. The detection limit was 1 CFU/mL and the countable range was 30-300 CFU/mL.

For the determination of the phenotypic resistance of *Pseudomonas aeruginosa* to antibiotics, the disk diffusion method was used, in accordance with the methodology described by the National Institute of Health (INS)-MINSA of 2002 ^18^ and by the Clinical and Laboratory Standards Institute ^19^. The antibiotics tested were: ceftazidime (CAZ-30µg), cefepime (FEP-30µg), gentamicin (GM-10µg), amikacin (AMK-30µg), imipenem (IPM-10µg), meropenem (MEM-10µg), cefoperazone-sulbactam (SUL-30µg), aztreonam (AZT-30µg), ciprofloxacin (CIP-5µg) and norfloxacin (NX-10µg), which were acquired commercially. For the determination of resistant isolates, the terminology proposed by Magiorakos A. *et al.*^20^ was followed regarding the definition of different multidrug-resistant (MDR), extensively drug-resistant (XDR), and pandrug-resistant (PDR) bacteria.

Quality controls were implemented at all stages of the microbiological analyses. As medium, reagent, and positive controls, the reference strains *Pseudomonas aeruginosa* ATCC 27853 and *E. coli* ATCC 25992 were used; as negative controls, strains of *Staphylococcus aureus* ATCC 6538, *Enterobacter aerogenes* ATCC 13048, and *Enterobacter cloacae* ATCC 13047 were used for the multiple-tube fermentation technique.

*Enumeration of coliforms and Escherichia coli*

Primary isolation was performed using double-strength lauryl sulfate broth supplemented with 0.01% bromocresol purple, inoculated and incubated in a series of 10 tubes for 24-48 hours at 35 °C. Subsequently, the color change of the medium from purple to yellow due to acidification of the medium was observed; with suspicion of coliforms, inoculation continued in brilliant green bile broth for 48 hours at 35 °C, to confirm total coliforms, and EC broth and tryptone water for 24 hours at 44.5 °C, for thermotolerant coliforms and presence of *Escherichia coli*, respectively. The indole test was performed with Kovács' reagent from the tryptone water ^15^.

*Enumeration of Pseudomonas aeruginosa*

The detection of *Pseudomonas aeruginosa* was performed using an adaptation of the conventional method, integrating its isolation from the lauryl sulfate broth tubes supplemented with 0.01% bromocresol purple used in the MPN technique for coliforms. Those tubes with growth, turbidity, or surface film formation without color change were selected, which were additionally incubated for 24 h at 36 °C. Subsequently, they were plated on cetrimide agar and incubated for up to 48 h at 36 °C. Presumptive colonies were subcultured on King B agar and incubated for 3-5 days at 35 °C, evaluating fluorescence production with a long-wave ultraviolet lamp (365 nm). Presumptive *Pseudomonas aeruginosa* colonies were confirmed by ammonia production from acetamide (Nessler's reagent) and a positive oxidase test. Positive colonies in these three tests were considered *Pseudomonas aeruginosa*, in accordance with standard 16266:2006 ^17^.

*Quantification of heterotrophic bacteria*

The 9215 Heterotrophic Plate Count method from APHA, AWWA, and WEF ^15^ was followed, where serial dilutions were performed from the samples, and 0.1 mL per dilution was plated in duplicate on Petri dishes with Plate Count agar, and incubated for 48 hours at 35 °C ^15^.

### Statistical analysis

The unit of analysis was the individual water sample (n=145), on which absolute frequencies, percentages, and distributions of the evaluated microbiological indicators were calculated. Samples were collected from 16 sampling points, including 9 fixed points evaluated monthly throughout the study period, which generated repeated measurements at the same points and, therefore, a potential dependence among observations. In contrast, samples from aircraft corresponded to distinct units on each occasion, being considered independent of each other.

Given that the study had a descriptive approach, the results are presented at the sample level, without performing inferential analyses or modeling intra-point correlation. Data processing was performed using Microsoft Excel.

### Ethical Aspects

The present study was carried out within the framework of the health surveillance competencies of the Dirección de Sanidad Aérea Internacional, an agency attached to the Dirección Ejecutiva de Sanidades Internacionales of the Dirección Regional de Salud del Callao, and has the approval of the corresponding institutional Ethics Committee (Official Letters: Certificate No. 001-2024 and No. 041-2024-Comité de Ética/UI/DIRESA/Callao).

## RESULTS

145 drinking water samples from airport terminal taps, restaurants, water supply companies, wells, and aircraft were analyzed during 2024. Sample classification as suitable or unsuitable was performed exclusively based on the microbiological parameters established in DS N.° 031-2010-SA, without considering the presence of *Pseudomonas aeruginosa*, which was evaluated as a complementary indicator. 50.3% (73/145) were classified as unsuitable for human consumption (table 1). Regarding the microbiological indicators evaluated, the presence of total coliforms was detected in 12.4% (18/145) of the samples, thermotolerant coliforms in 9.7% (14/145), and *E. coli* in 2.1% (3/145). Heterotrophic bacteria exceeded the maximum permissible limit in 45.5% of the samples (66/145). Additionally, *P. aeruginosa* was isolated in 55.9% (81/145) of the total analyzed.

**Table 1:** Frequency of non-compliance with established microbiological parameters for human consumption water in 145 samples analyzed during 2024.

Microbiological parameter	Samples that exceed the maximum permissible limit (n/N)	%
Total Coliforms	18/145	12.4
Thermotolerant coliforms	14/145	9.7
*Escherichia coli*	3/145	2.1
Heterotrophic bacteria	66/145	45.5
Unsuitable samples^a^	73/145	50.3

a Samples that failed to comply with at least one of the microbiological parameters established in DS N.° 031-2010-SA.

The values corresponding to each parameter are not mutually exclusive, so their sum may be greater than the total number of unsuitable samples.

Methods used: total coliforms, thermotolerant coliforms and *E. coli*: multiple-tube fermentation technique (MPN), APHA, AWWA and WEF 9221 B, C, E and G ^15^. Heterotrophic bacteria: plate count, APHA, AWWA and WEF 9215 B.

**Figure 1.**
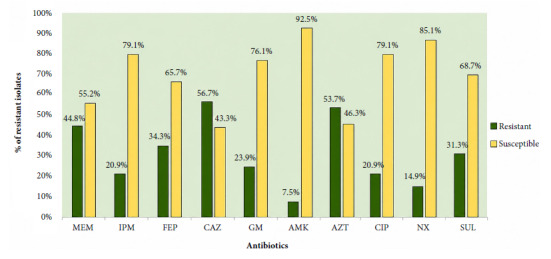
Percentages of in vitro resistance and sensitivity to antibiotics of *Pseudomonas aeruginosa* isolated from water for human consumption from the old terminal of Jorge Chavez International Airport, 2024.

Frequent detections of *P. aeruginosa* were observed in national and international flight aircraft, with concentrations >16 NMP/100 mL in several months. Heterotrophic bacterial counts ranged from 34 to 2560 CFU/mL, with higher values during the third quarter. Additionally, isolated episodes of coliforms >23 NMP/100 mL and punctual detection of *E. coli* were recorded (table 2). When analyzing the distribution of microbiological contamination by type of sampling point, differences were identified among the components of the water system. In the airport terminal taps, episodic increases of coliforms were observed, with maximum values of up to 9.2 NMP/100 mL and punctual detections of *E. coli*. In restaurants, transient episodes were recorded with concentrations >23 NMP/100 mL of total and thermotolerant coliforms. In wells, dispensers, and tanker trucks, low values predominated during most of the study period, although occasional increases >23 NMP/100 mL were observed. Regarding *P. aeruginosa*, the terminal taps showed concentrations >16 NMP/100 mL in several months, while in wells and supplier companies, concentrations were generally ≤2.2 NMP/100 mL, with transient increases reaching 16 and >16 NMP/100 mL (table 3). For the phenotypic evaluation of antimicrobial resistance, 67 *P. aeruginosa* isolates were analyzed. 38.8% (26/67) were classified as multidrug-resistant (MDR), 11.9% (8/67) as extensively drug-resistant (XDR), and no pandrug-resistant (PDR) isolates were identified. The remaining 49.3% showed extended resistance (table 4). Variability was observed in susceptibility profiles, with predominant resistance to aminoglycosides and some cephalosporins, while most isolates retained susceptibility to carbapenems (figure 1).

**Table 2:** Individual microbiological results of 13 samples of water for human consumption obtained from commercial aircraft from national and international flights during 2024.

Airline	Month	Total coliforms MPN/100mL	Thermotolerant coliforms MPN/100mL	***E. coli* MPN/100mL**	Heterotrophic bacteria CFU/mL	***P. aeruginosa* MPN/100mL**
A1 International	January	<1.1	<1.1	<1.1	133	>16
A2 National	February	>23	>23	<1.1	675	>16
A1 National	March	<1.1	<1.1	<1.1	67	>16
A3 National	April	<1.1	<1.1	<1.1	95	>16
A1 Nacional	May	<1.1	<1.1	<1.1	112	>16
A1 International	June	<1.1	<1.1	<1.1	98	>16
A1 Nacional	Julio	<1.1	<1.1	<1.1	34	<2.2
A3 National	August	>23	>23	12	2560	>16
A3 International	September	>23	16	<1.1	835	>16
A1 National	October	<1.1	<1.1	<1.1	51	5.1
A3 National	November	<1.1	<1.1	<1.1	420	2.2
A3 International	December	3.6	3.6	<1.1	730	>16
A1 International	December	1.1	1.1	<1.1	610	>16

The 13 samples presented correspond to the samples taken from the taps of commercial aircraft among the 145 human consumption water samples analyzed during the study period. Each row corresponds to an independent sample obtained from a commercial aircraft (8 domestic flights and 5 international flights).

Values expressed as "<" and ">" correspond to the quantification limits of the Most Probable Number (MPN) method used for the determination of coliforms and *P. aeruginosa*.

The airlines were coded (A1, A2 and A3) to preserve the confidentiality of the information.

**Table 3:** Monthly results of *Pseudomonas aeruginosa* in human consumption water samples obtained in airport facilities and associated supply systems during 2024.

Sample Code		**Months of sampling and results for *P. aeruginosa* (MPN/100mL)**											
		En.	Feb.	Mar.	Apr.	May.	Jun.	Jul.	Ag.	Set.	Oct.	Nov.	Dec.
Taps of the airport terminal													
	GT-01 - Dining room of the internal administrative facility (1st level)	<2,2	2,2	<2,2	<2,2	9.2	>16	>16	5.1	2.2	>16	<2,2	>16
	GT-02 - Bathroom located next to the administrative facility (1st level)	<2,2	9.2	<2,2	<2,2	<2,2	<2,2	<2,2	<2,2	2.2	<2,2	2.2	>16
	GT-03 - Bathroom next to the national area food court (2nd level)	5,1	9.2	<2,2	<2,2	<2,2	>16	>16	<2,2	9.2	<2,2	<2,2	>16
	GT-04 - Restroom in front of the lost and found area (1st level)	2,2	9.2	<2,2	<2,2	<2,2	>16	>16	<2,2	<2,2	<2,2	2.2	2.2
Well of the airport terminal													
	PZ-01 - 1st bathroom tap near well No. 1	2,2	2,2	<2,2	<2,2	<2,2	2.2	2,2	2,2	<2,2	<2,2	2.2	2,2
Restaurant taps													
	GR-01 - Kitchen faucet national area (2nd level)	>16	<2,2	<2,2	<2,2	<2,2	>16	2.2	>16	<2,2	<2,2	<2,2	<2,2
	GR-02 - Kitchen faucet national zone (2nd level)	16	<2,2	<2,2	<2,2	2,2	>16	2.2	>16	<2,2	<2,2	<2,2	<2,2
	GR-03 - Bar tap, international area (2nd level)	<2,2	<2,2	<2,2	<2,2	2.2	2,2	>16	2,2	>16	<2,2	>16	>16
	GR-04 - Kitchen faucet international zone (2nd level)	5.1	<2,2	<2,2	5.1	5.1	5,1	>16	5,1	>16	>16	>16	>16
Tanker trucks from supplier companies													
	CI-01 - Tanker truck from supplier company A	<2,2	NS	NS	NS	16	<2,2	NS	NS	2.2	NS	NS	NS
	CI-02 - Tanker truck of supplier company A	5.1	NS	NS	NS	>16	<2,2	NS	NS	2.2	NS	NS	NS
	CI-03 - Tank truck of supplier company B	NS	<2,2	NS	16	NS	NS	<2.2	NS	NS	<2,2	NS	NS
	CI-04 - Tank truck from supplier company C	NS	NS	<2,2	NS	NS	NS	NS	9.2	NS	NS	<2,2	>16
Well “air side” of provider company													
	PZ-02 - Well air side of supplier company B	NS	2.2	NS	<2,2	NS	NS	>16	NS	NS	<2,2	NS	NS
Water dispenser for human consumption													
	SR-01 - Supplier of empresa proveedora C	NS	NS	<2,2	NS	NS	NS	NS	9.2	NS	NS	<2,2	>16

NS: not sampled. <2.2 MPN/100 mL: result below the method's detection limit. >16 MPN/100 mL: result above the method's upper detection limit.

The table presents individual results obtained at the sampling points evaluated during 2024.

Fixed points were sampled monthly, while the points associated with supplier companies were evaluated according to the established sampling plan.

The samples for each human consumption water supplier company A, B and C were taken 4 times a year.

Methods used: the Modified Most Probable Number (MPN) method by Ramírez Z. ^9^, Guevara J. et al. ^16^ was employed, and confirmation criteria according to ISO 16266 ^17^ were followed.

**Table 4:** Distribution of *Pseudomonas aeruginosa* isolates evaluated for antimicrobial susceptibility according to resistance profile (MDR, XDR and Non-MDR/XDR) and sampling sites (n=67).

Sampling points / Laboratory code			Isolates evaluated (n)	MDR (n)	XDR (n)	No MDR/XDR (n)
Airport terminal taps						
		GT-01	5	2	2	1
		GT-02	1	1	0	0
		GT-03	4	1	0	3
		GT-04	4	2	0	2
Wells						
		PZ-01	5	2	1	2
		PZ-02	7	4	1	2
Tanker trucks						
		CI-01	1	0	0	1
		CI-02	1	0	0	1
		CI-03	5	1	0	4
		CI-04	2	2	0	0
Supplier						
		SR-01	2	1	0	1
Restaurant taps						
		GR-01	3	2	0	1
		GR-02	3	2	0	1
		GR-03	6	1	2	3
		GR-04	8	3	2	3
Commercial flights						
	National flights		6	0	0	6
		AE-N01	1	0	0	1
		AE-N02	3	0	0	3
		AE-N03	1	0	0	1
		AE-N04	1	0	0	1
	International flights		4	2	0	2
		AE-I01	2	0	0	2
		AE-I02	1	1	0	0
		AE-I03	1	1	0	0
Total, n (%)			67 (100)	26 (38.8)	8 (11.9)	33 (49.3)

MDR: multidrug-resistant. XDR: extensively drug-resistant. No MDR/XDR: isolates that do not meet the criteria for multidrug resistance nor extreme resistance.

The number of isolates included in the antimicrobial susceptibility tests does not necessarily correspond to the number of positive months registered for each sampling point. The isolates were obtained during the study period, preserved, and subsequently reactivated for their joint processing by antibiogram. Consequently, a sampling point could provide more than one isolate or a smaller number of isolates than the total number of positive isolates registered.

MEM: meropenem, IPM: imipenem, FEP: cefepime, CAZ: ceftazidime, GM: gentamicin, AMK: amikacin, AZT: aztreonam, CIP: ciprofloxacin, NX: norfloxacin, SUL: cefoperazone-sulbactam.

## DISCUSSION

International air transport facilitates the global dissemination of resistant bacteria and other pathogens between geographical regions ^21^^,^^22^. In a study conducted by Handschuh H. et al. (2017), it was demonstrated that the predominant gammaproteobacteria in the water from aircraft lavatories and galleys belong to the genus *Pseudomonas*, acting as potential reservoirs of antimicrobial resistance ^21^. Likewise, the presence of various opportunistic pathogens was identified, including *P. aeruginosa*, with the capacity to colonize aircraft water systems ^23^^,^^24^. In this context, the identification of multidrug-resistant (MDR) and extensively drug-resistant (XDR) strains in airport supply systems suggests that these infrastructures could function as environmental reservoirs, with the potential for cross-border spread ^25^.

55.2% of the isolates showed resistance to at least one antimicrobial, with higher levels against ceftazidime (56.7%) and aztreonam (53.7%), followed by cefepime (34.3%). Resistance to carbapenems such as meropenem (44.8%) and imipenem (20.9%) is particularly relevant due to its association with high morbidity and mortality and therapeutic limitation ^14^^,^^25^. The resistance observed against aminoglycosides and quinolones, as well as cefoperazone-sulbactam, suggests the involvement of multiple intrinsic and acquired mechanisms described in *P. aeruginosa*, such as the production of beta-lactamases, the overexpression of efflux pumps, and the decrease in outer membrane permeability ^24^. These findings coincide with international reports on resistant *P. aeruginosa* in drinking water and critical infrastructure, although specific evidence in airports remains limited ^25^. Likewise, coliform indicators and heterotrophic bacteria show vulnerabilities of the water system and conditions that could favor biofilm formation and persistence of *P. aeruginosa* in the distribution network.

In this context, it is important to interpret with caution the proportion of samples classified as “unsuitable” (50.3%), given that this reflects the non-compliance with normative microbiological criteria in a set of heterogeneous points of the water system, evaluated under a risk-based sampling approach. When considering the stratification by type of sampling point, differentiated patterns are evidenced, where internal distribution systems and intensive-use environments (such as terminal faucets, restaurants, and aircraft) show greater variability and contamination episodes, in contrast with sources such as wells and supplier companies, which showed greater microbiological stability. In this regard, the findings suggest the coexistence of sanitary risks associated with normative non-compliance and operational contamination phenomena linked to the inherent dynamics of complex water distribution systems in airport infrastructures.

Although *P. aeruginosa* is not part of the basic microbiological compliance criteria for routine water potability assessment according to current national regulations, its presence is considered in the WHO guidelines for drinking water quality as a relevant indicator in specific contexts, particularly in distribution systems where it can indicate biofilm formation, environmental colonization, and possible operational contamination, especially in internal networks and complex distribution systems such as those in airports. In this regard, the high frequency observed could be related to this microorganism's ability to survive in environments with low nutrient availability. Its presence may represent a potential risk for the susceptible population. However, its relevance lies in its nature as an opportunistic pathogen, especially in vulnerable populations, as well as its role as a reservoir of antimicrobial resistance, which justifies its inclusion as a complementary indicator in health surveillance contexts in highly complex infrastructures such as airports.

Investigations in European and Asian airports have documented similar findings, linking contamination to internal distribution networks and supply vehicles ^26^^-^^29^. Although there is no conclusive evidence of direct transmission of infections from water consumption on aircraft ^23^, underreporting may be influenced by the incubation period and the high mobility of passengers ^27^.

In Latin America, local studies have reported *P. aeruginosa* resistance in water sources, although with a lower frequency of resistance than that observed in this study ^16^^,^^28^. This underscores the importance of local surveillance, given that resistance patterns vary according to geographical context. Globally, carbapenem-resistant *P. aeruginosa* constitutes one of the main health threats, ranking among the pathogens with the highest attributable mortality burden ^4^. Risk models have shown that intestinal colonization after ingesting contaminated water is low under usual conditions but increases significantly with high bacterial concentrations and concomitant antibiotic use ^30^. In immunocompromised individuals, this risk can be clinically relevant. The persistence of *P. aeruginosa* in drinking water is associated with its ability to form biofilms, tolerate residual chlorine, and adapt to environments with low nutritional content ^29^, which explains its survival in distribution networks. The detection of MDR and XDR strains in an international airport, a highly connected environment, extends the discussion beyond traditional surveillance of transmissible diseases, incorporating antimicrobial resistance as a critical biosecurity component in these areas.

The study's limitations include restricted access to certain critical points of the water system and the health risk-based sampling approach. The selection of sampling points was non-probabilistic and prioritized components considered critical for health surveillance, so the results cannot be directly extrapolated to the entire distribution network. Additionally, the number of samples obtained was not uniform among the different points evaluated due to operational differences and the nature of the system components, generating an asymmetric distribution of observations. Likewise, the presence of repeated measurements at fixed points over time introduces dependence among observations, which was not statistically modeled due to the descriptive approach of the study, so the results should be interpreted at the sample level and not as completely independent observations. The absence of molecular characterization of the isolates constitutes a relevant limitation, since it does not allow determining whether the recurrent detections of *P. aeruginosa* correspond to the persistence of the same bacterial clone within the water system or to independent contamination events. Consequently, although the findings suggest favorable conditions for the persistence of this microorganism, they do not allow confirming specific mechanisms of maintenance or dispersion within the network. Furthermore, no direct evaluations of biofilm formation were performed, considered one of the main mechanisms of persistence of *P. aeruginosa* in water distribution systems, so its involvement in the observed findings could not be demonstrated. Finally, the study was conducted in the infrastructure of the old airport terminal, so the results cannot be directly extrapolated to the new airport infrastructure. Despite these limitations, the findings support the need to strengthen microbiological surveillance at international entry points and implement preventive strategies for water quality management, in accordance with the principles of the International Health Regulations and with specific regulations applicable to water systems on aircraft ^31^^,^^32^. Furthermore, emerging environmental surveillance approaches, including wastewater monitoring on aircraft and at airports, have demonstrated usefulness for early pathogen detection and could complement traditional health surveillance systems ^33^^,^^34^.

In conclusion, the results showed a high frequency of non-compliance with the microbiological parameters established for human consumption water and a recurrent presence of *P. aeruginosa* with antimicrobial resistance profiles in different components of the airport water system, including aircraft. These findings suggest the existence of conditions that could favor the persistence and circulation of resistant bacteria at certain points of the system. Likewise, they constitute a baseline for strengthening microbiological and antimicrobial resistance surveillance in water systems associated with international entry points, as well as for the development of future research aimed at clarifying the mechanisms of persistence, dispersion, and resistance of these microorganisms in airport environments.

## References

[1] Organismo Supervisor de la Inversión en Infraestructura de Transporte de Uso Público (2024). Informe de desempeño 2023: Aeropuerto Internacional Jorge Chávez.

[2] Organización Mundial de la Salud (2017). Guías para la calidad del agua de consumo humano: cuarta edición que incorpora la primera adenda.

[3] Organización Mundial de la Salud (2006). Guías para la calidad del agua potable: primer apéndice a la tercera edición.

[4] GBD 2021 Antimicrobial Resistance Collaborators (2024). Global burden of bacterial antimicrobial resistance 1990-2021: a systematic analysis with forecasts to 2050. Lancet.

[5] Salam MA, Al-Amin MY, Salam MT, Pawar JS, Akhter N, Rabaan AA (2023). Antimicrobial Resistance A Growing Serious Threat for Global Public Health. Healthcare (Basel).

[6] Frost I, Van Boeckel T, Pires J, Craig J, Laxminarayan R (2019). Global geographic trends in antimicrobial resistance the role of international travel. J Travel Med.

[7] Ahmed W, Bivins A, Simpson L, Bertsch PM, Ehret J, Hosegood I (2022). Wastewater surveillance demonstrates high predictive value for COVID-19 infection on board repatriation flights to Australia. Environ Int.

[8] (2010). Reglamento de la Calidad del Agua para Consumo Humano, Decreto Supremo N.° 031-2010-SA. Diario El Peruano.

[9] Ramírez-Zapata E, Delgado-Portales R, Moscosa-Santillán M, De la Cruz-Martínez A, Loredo-Becerra A (2023). Detección e incidencia de Pseudomonas en muestras de agua potable. Acta de Ciencia en Salud.

[10] (2008). Norma Sanitaria que establece los criterios microbiológicos de calidad sanitaria e inocuidad para los alimentos y bebidas de consumo humano, Resolución Ministerial N.° 591-2008-MINSA. Diario El Peruano.

[11] (2016). Determinación del índice de Calificación Sanitaria de las Piscinas Públicas y Privadas de uso Colectivo, Resolución Ministerial N.° 527-2016-MINSA. Diario Oficial El Peruano.

[12] World Health Organization (2024). WHO bacterial priority pathogens list, 2024: bacterial pathogens of public health importance to guide research, development and strategies to prevent and control antimicrobial resistance.

[13] Torres Y (1991). Utilización de Pseudomonas aeruginosa como indicador de contaminación en el agua de tanques y cisternas. Boletín de Lima.

[14] Rodríguez S, Asmundis C, Ayala M, Arzú O (2018). Presencia de indicadores microbiológicos en agua para consumo humano en San Cosme (Corrientes, Argentina). Rev Vet.

[15] American Public Health Association American Water Works Association. Water Environment Federation (2023). Standard methods for the examination of water and wastewater.

[16] Álvarez Guevara J (2016). Control de calidad microbiológica del agua para consumo.

[17] International Organization for Standardization (2006). ISO 16266-2:2006. Water quality. Detection and enumeration of Pseudomonas aeruginosa. Part 2: method by membrane filtration.

[18] Instituto Nacional de Salud (2002). Manual de procedimientos para la prueba de sensibilidad antimicrobiana por el método de disco difusión. Serie de normas técnicas N.° 30.

[19] Clinical and Laboratory Standards Institute (2009). Performance standards for antimicrobial susceptibility testing: nineteenth informational supplement (M100-S19).

[20] Magiorakos AP, Srinivasan A, Carey RB, Carmeli Y, Falagas ME, Giske CG (2012). Multidrug-resistant, extensively drug-resistant and pandrug-resistant bacteria an international expert proposal for interim standard definitions for acquired resistance. Clin Microbiol Infect.

[21] Handschuh H, Ryan MP, O'Dwyer J, Adley CC (2017). Assessment of the bacterial diversity of aircraft water identification of the frequent fliers. PLoS ONE.

[22] De Bentzmann S, Plésiat P (2011). The Pseudomonas aeruginosa opportunistic pathogen and human infections. Environ Microbiol.

[23] Knight ME, Farkas K, Wade M, Webster G, Pass DA, Perry W (2025). Wastewater-based analysis of antimicrobial resistance at UK airports evaluating the potential opportunities and challenges. Environ Int.

[24] Harald H (2015). Bacteria that travel the quality of aircraft water. Int J Environ Res Public Health.

[25] Rai SB, Thiruvengadam V, Hendersen A (2013). Contamination of Potable Water at Penang International Airport - a Disaster Averted. Outbreak Surveill Investig Rep.

[26] Public Health Laboratory Service. Association of Port Health Authorities (2003). The microbiological quality of water onboard aircraft.

[27] Mangili A, Gendreau MA (2005). Transmission of infectious diseases during commercial air travel. Lancet.

[28] Correa Rivas KA, Bravo Torrealba MV, Silva Alvarado RA, Montiel M (2015). Susceptibilidad a antibióticos de Pseudomonas aeruginosa aislada de agua de consumo humano de la comunidad Santa Rosa de Agua, Maracaibo, estado Zulia. Rev Soc Venez Microbiol.

[29] Villegas Blanco M, Prado López I, Ortega Gónzalez M, Zhurbenko R (2012). Identificación de Pseudomonas aeruginosa empleando el método del número más probable. Rev Perú Epidemiol.

[30] Buck AC, Cooke EM (1969). The fate of ingested Pseudomonas aeruginosa in normal persons. J Med Microbiol.

[31] Organización Mundial de la Salud (2005). Reglamento Sanitario Internacional (2005).

[32] U.S. Environmental Protection Agency (2009). Aircraft Drinking Water Rule (ADWR).

[33] Li J, Hosegood I, Powell D, Tscharke B, Lawler J, Thomas KV (2023). A global aircraft-based wastewater genomic surveillance network for early warning of future pandemics. Lancet Glob Health.

[34] Tay M, Lee B, Muhammad Hafiz I, Yam J, Dzulkhairul M, Yew-Hoong K (2024). Utility of aircraft and airport wastewater for monitoring multiple pathogens including SARS-CoV-2 variants. J Travel Med.

