# Supplementary material for: Microbiological quality and antimicrobial resistance of Pseudomonas aeruginosa in drinking water systems of the old terminal of Jorge Chávez International Airport: potential role as environmental reservoir
Source: Rev Peru Med Exp Salud Publica. 2026 Jun 30;43(2):207–16. doi: 10.17843/rpmesp.2026.432.15160 (PMC13456917; doi:10.17843/rpmesp.2026.432.15160)
Supplement: Supplementary file 2 [file rpmesp-43-02-15160-s001.docx]

**MATERIAL SUPLEMENTARIO**

**Material suplementario 1.** Límites máximos permisibles de parámetros microbiológicos y parasitológicos.

| **Parámetros** | **Unidad de medida** | **Límite máximo permisible** |
| --- | --- | --- |
| Bacterias Coliformes totales | UFC/100 mL a 35°C | Ausencia (*) |
| *E.coli* | UFC/100 mL a 44.5°C | Ausencia (*) |
| Bacterias coliformes termotolerantes o fecales | UFC/100 mL a 44.5°C | Ausencia (*) |
| Bacterias heterotróficas | UFC/mL a 35°C | 500 |
| Huevos y larvas de helmintos, quistes y ooquistes de protozoarios patógenos. | N° Org/L | Ausencia |
| Virus | UFC/mL | Ausencia |
| Organismos de vida libre, como algas, protozoarios, copépodos, rotíferos, nematodos en todos sus estadios evolutivos | N° Org/L | Ausencia |

UFC = Unidad formadora de colonias

(*) En caso de analizar por la técnica de tubos múltiples = <1.8/100 mL. NMP.

*Fuente:* MINSA (2010) Reglamento de la Calidad para el agua para consumo humano.

**Material suplementario 2.** Ubicación y descripción de los sitios de toma de muestra de agua de consumo humano dentro del Aeropuerto Internacional 2024.

| **Código de laboratorio**  **(Muestra)** | **Ubicación^1^** | **Sistema de aducción** | **Descripción** | **Observaciones** |
| --- | --- | --- | --- | --- |
| **SH-0DSAI** | Dirección de sanidad aérea internacional | Red de distribución interno | Zona del comedor de la DSAI | Grifo de la cocina dentro de DSAI |
| **SH-01J** | Terminal aeroportuario | Red de distribución interno | Baño ubicado al lado de DSAI | Grifo 1er nivel |
| **SH-02J** | Terminal aeroportuario | Red de distribución interno | Baño al lado del patio de comidas y frente al control de seguridad | Grifo 2do nivel |
| **SH-07J** | Terminal aeroportuario | Red de distribución interno | Baño frente al área de objetos perdidos | Grifo 1er nivel |
| **PZO-LAP** | Frente al pozo N°01 de la terminal aeroportuaria | Red de distribución interno | Pozo | Grifo del baño que es la primera salida del pozo N°01 |
| **TLM-CA10** | Dentro de la pista de aterrizaje | Mangueras de transferencia | Carros cisterna | Empresa A proveedora de agua potable |
| **TLM-CA11** | Dentro de la pista de aterrizaje | Mangueras de transferencia | Carros cisterna | Empresa A proveedora de agua potable |
| **SAA-TA-01** | Dentro de la pista de aterrizaje | Mangueras de transferencia | Carros cisterna | Empresa B proveedora de agua potable |
| **PZO-SAA** | Dentro de la pista de aterrizaje | Bombeo | Pozo | Empresa B proveedora de agua potable |
| **SRT-SWS** | Dentro de la pista de aterrizaje | Mangueras de transferencia | Carros cisterna | Empresa C proveedora de agua potable |
| **SGP-CA-1** | Dentro de la pista de aterrizaje | Bombeo | Surtidor de agua para consumo humano | Empresa C proveedora de agua potable |
| **TANT1-01** | Patio de comidas | Red de distribución interno | Grifo de cocina  (restaurante) | Grifo 2do nivel |
| **LACH1-01** | Patio de comidas | Red de distribución interno | Grifo de cocina (restaurante) | Grifo 2do nivel |
| **LANL-01** | Sala de espera zona internacional | Red de distribución interno | Grifo de bar  (restaurante) | Grifo 2do nivel |
| **BON-01** | Sala de espera zona internacional | Red de distribución interno | Grifo de cocina  (restaurante) | Grifo 2do nivel |

**Material suplementario 3**. Resultados microbiológicos mensuales de muestras de agua para consumo humano: Coliformes totales, fecales o termotolerantes, *Escherichia coli* y bacterias heterotróficas.

| **Códigos de muestra** | **Meses de muestreo** | | | | | | | | | | | |
| --- | --- | --- | --- | --- | --- | --- | --- | --- | --- | --- | --- | --- |
| **Grifos de la terminal aeroportuaria** | **ENE** | **FEB** | **MAR** | **ABR** | **MAY** | **JUN** | **JUL** | **AGO** | **SEP** | **OCT** | **NOV** | **DIC** |
| **SH-0DSAI** | **Grifo del comedor de la DSAI (1er nivel)** | | | | | | | | | | | |
| **Concentración de CT NMP/100mL** | **<1,1** | **<1,1** | **<1,1** | **<1,1** | **<1,1** | **<1,1** | **<1,1** | **<1,1** | **<1,1** | **<1,1** | **<1,1** | **<1,1** |
| **Concentración de CF**  **NMP/100mL** | **<1,1** | **<1,1** | **<1,1** | **<1,1** | **<1,1** | **<1,1** | **<1,1** | **<1,1** | **<1,1** | **<1,1** | **<1,1** | **<1,1** |
| **Concentración de *E. coli***  **NMP/100mL** | **<1,1** | **<1,1** | **<1,1** | **<1,1** | **<1,1** | **<1,1** | **<1,1** | **<1,1** | **<1,1** | **<1,1** | **<1,1** | **<1,1** |
| **Concentración de Bact.Het**  **UFC/mL (x10^2^)** | **3,95** | **20** | **3,50** | **0,10** | **6,78** | **5,8** | **34** | **1,58** | **0,85** | **3,40** | **0,02** | **0,10** |
| **SH-01J** | **Grifo del baño ubicado al lado de DSAI (1er nivel)** | | | | | | | | | | | |
| **Concentración de CT NMP/100mL** | **<1,1** | **<1,1** | **<1,1** | **<1,1** | **<1,1** | **<1,1** | **<1,1** | **<1,1** | **<1,1** | **<1,1** | **<1,1** | **1,1** |
| **Concentración de CF**  **NMP/100mL** | **<1,1** | **<1,1** | **<1,1** | **<1,1** | **<1,1** | **<1,1** | **<1,1** | **<1,1** | **<1,1** | **<1,1** | **<1,1** | **1,1** |
| **Concentración de *E. coli***  **NMP/100mL** | **<1,1** | **<1,1** | **<1,1** | **<1,1** | **<1,1** | **<1,1** | **<1,1** | **<1,1** | **<1,1** | **<1,1** | **<1,1** | **<1,1** |
| **Concentración de Bact.Het**  **UFC/mL (x10^2^)** | **1,40** | **26** | **1,50** | **2** | **3,31** | **11** | **10** | **4,20** | **2,50** | **1,10** | **0,13** | **1,25** |
| **SH-02J** | **Baño al lado del patio de comidas zona nacional (2do nivel)** | | | | | | | | | | | |
| **Concentración de CT NMP/100mL** | **<1,1** | **<1,1** | **<1,1** | **<1,1** | **<1,1** | **16** | **<1,1** | **<1,1** | **<1,1** | **<1,1** | **<1,1** | **<1,1** |
| **Concentración de CF**  **NMP/100mL** | **<1,1** | **<1,1** | **<1,1** | **<1,1** | **<1,1** | **2,2** | **<1,1** | **<1,1** | **<1,1** | **<1,1** | **<1,1** | **<1,1** |
| **Concentración de *E. coli***  **NMP/100mL** | **<1,1** | **<1,1** | **<1,1** | **<1,1** | **<1,1** | **<1,1** | **<1,1** | **<1,1** | **<1,1** | **<1,1** | **<1,1** | **<1,1** |
| **Concentración de Bact.Het**  **UFC/mL (x10^2^)** | **0,08** | **28** | **6** | **2,46** | **4,10** | **7,4** | **28** | **5,5** | **7,8** | **0,8** | **0,68** | **0,90** |
| **SH-07J** | **Baño frente al área de objetos perdidos (1er nivel)** | | | | | | | | | | | |
| **Concentración de CT NMP/100mL** | **9,2** | **<1,1** | **<1,1** | **<1,1** | **<1,1** | **6,9** | **<1,1** | **<1,1** | **<1,1** | **<1,1** | **<1,1** | **<1,1** |
| **Concentración de CF**  **NMP/100mL** | **9,2** | **<1,1** | **<1,1** | **<1,1** | **<1,1** | **6,9** | **<1,1** | **<1,1** | **<1,1** | **<1,1** | **<1,1** | **<1,1** |
| **Concentración de *E. coli***  **NMP/100mL** | **3,6** | **<1,1** | **<1,1** | **<1,1** | **<1,1** | **<1,1** | **<1,1** | **<1,1** | **<1,1** | **<1,1** | **<1,1** | **<1,1** |
| **Concentración de Bact.Het**  **UFC/mL (x10^2^)** | **2,53** | **30** | **3** | **4,5** | **5** | **8,1** | **11** | **6.3** | **9,4** | **0,05** | **0,06** | **0,04** |
| **Pozo de la terminal aeroportuaria** | **ENE** | **FEB** | **MAR** | **ABR** | **MAY** | **JUN** | **JUL** | **AGO** | **SEP** | **OCT** | **NOV** | **DIC** |
| **PZO-LAP** | **Grifo del 1er baño cercano al pozo N°01** | | | | | | | | | | | |
| **Concentración de CT NMP/100mL** | **<1,1** | **<1,1** | **<1,1** | **<1,1** | **<1,1** | **<1,1** | **<1,1** | **<1,1** | **<1,1** | **<1,1** | **<1,1** | **<1,1** |
| **Concentración de CF**  **NMP/100mL** | **<1,1** | **<1,1** | **<1,1** | **<1,1** | **<1,1** | **<1,1** | **<1,1** | **<1,1** | **<1,1** | **<1,1** | **<1,1** | **<1,1** |
| **Concentración de *E. coli***  **NMP/100mL** | **<1,1** | **<1,1** | **<1,1** | **<1,1** | **<1,1** | **<1,1** | **<1,1** | **<1,1** | **<1,1** | **<1,1** | **<1,1** | **<1,1** |
| **Concentración de Bact.Het**  **UFC/mL (x10^2^)** | **30,1** | **25** | **15** | **9,4** | **32,1** | **40** | **8,1** | **5,7** | **4,20** | **0,29** | **0,26** | **1,02** |
| **Grifos de restaurantes** | **ENE** | **FEB** | **MAR** | **ABR** | **MAY** | **JUN** | **JUL** | **AGO** | **SEP** | **OCT** | **NOV** | **DIC** |
| **TANT1-01** | **Grifo de cocina zona nacional (2do nivel)** | | | | | | | | | | | |
| **Concentración de CT NMP/100mL** | **<1,1** | **<1,1** | **<1,1** | **<1,1** | **<1,1** | **<1,1** | **<1,1** | **<1,1** | **<1,1** | **<1,1** | **<1,1** | **<1,1** |
| **Concentración de CF**  **NMP/100mL** | **<1,1** | **<1,1** | **<1,1** | **<1,1** | **<1,1** | **<1,1** | **<1,1** | **<1,1** | **<1,1** | **<1,1** | **<1,1** | **<1,1** |
| **Concentración de *E. coli***  **NMP/100mL** | **<1,1** | **<1,1** | **<1,1** | **<1,1** | **<1,1** | **<1,1** | **<1,1** | **<1,1** | **<1,1** | **<1,1** | **<1,1** | **<1,1** |
| **Concentración de Bact.Het**  **UFC/mL (x10^2^)** | **1,26** | **0,95** | **1,48** | **0,05** | **320** | **4,15** | **7,1** | **3,40** | **3,40** | **0,41** | **0,15** | **0,16** |
| **LACH1-01** | **Grifo de cocina zona nacional (2do nivel)** | | | | | | | | | | | |
| **Concentración de CT NMP/100mL** | **<1,1** | **<1,1** | **<1,1** | **<1,1** | **<1,1** | **<1,1** | **<1,1** | **<1,1** | **<1,1** | **<1,1** | **<1,1** | **<1,1** |
| **Concentración de CF**  **NMP/100mL** | **<1,1** | **<1,1** | **<1,1** | **<1,1** | **<1,1** | **<1,1** | **<1,1** | **<1,1** | **<1,1** | **<1,1** | **<1,1** | **<1,1** |
| **Concentración de *E. coli***  **NMP/100mL** | **<1,1** | **<1,1** | **<1,1** | **<1,1** | **<1,1** | **<1,1** | **<1,1** | **<1,1** | **<1,1** | **<1,1** | **<1,1** | **<1,1** |
| **Concentración de Bact.Het**  **UFC/mL (x10^2^)** | **2,36** | **0,46** | **2,25** | **0,42** | **299** | **3,55** | **5** | **4,5** | **5,20** | **0,35** | **0,08** | **0,12** |
| **LANL-01** | **Grifo del bar zona internacional (2do nivel)** | | | | | | | | | | | |
| **Concentración de CT NMP/100mL** | **<1,1** | **<1,1** | **<1,1** | **<1,1** | **<1,1** | **<1,1** | **<1,1** | **<1,1** | **<1,1** | **<1,1** | **<1,1** | **<1,1** |
| **Concentración de CF**  **NMP/100mL** | **<1,1** | **<1,1** | **<1,1** | **<1,1** | **<1,1** | **<1,1** | **<1,1** | **<1,1** | **<1,1** | **<1,1** | **<1,1** | **<1,1** |
| **Concentración de *E. coli***  **NMP/100mL** | **<1,1** | **<1,1** | **<1,1** | **<1,1** | **<1,1** | **<1,1** | **<1,1** | **<1,1** | **<1,1** | **<1,1** | **<1,1** | **<1,1** |
| **Concentración de Bact.Het**  **UFC/mL (x10^2^)** | **24,15** | **8,5** | **11,2** | **9** | **9,8** | **5,30** | **34,1** | **9,40** | **8,95** | **0,44** | **0,13** | **0,08** |
| **BON-01** | **Grifo de cocina zona internacional (2do nivel)** | | | | | | | | | | | |
| **Concentración de CT NMP/100mL** | **<1,1** | **<1,1** | **<1,1** | **<1,1** | **<1,1** | **<1,1** | **1,1** | **<1,1** | **<1,1** | **>23** | **1,1** | **<1,1** |
| **Concentración de CF**  **NMP/100mL** | **<1,1** | **<1,1** | **<1,1** | **<1,1** | **<1,1** | **<1,1** | **<1,1** | **<1,1** | **<1,1** | **>23** | **1,1** | **<1,1** |
| **Concentración de *E. coli***  **NMP/100mL** | **<1,1** | **<1,1** | **<1,1** | **<1,1** | **<1,1** | **<1,1** | **<1,1** | **<1,1** | **<1,1** | **3,6** | **<1,1** | **<1,1** |
| **Concentración de Bact.Het**  **UFC/mL (x10^2^)** | **21,35** | **10** | **6,72** | **10,1** | **15,8** | **258** | **28** | **7,8** | **10** | **0,6** | **0,44** | **0,41** |
| **Carros cisterna de las empresas proveedoras** | **ENE** | **FEB** | **MAR** | **ABR** | **MAY** | **JUN** | **JUL** | **AGO** | **SEP** | **OCT** | **NOV** | **DIC** |
| **TLM-CA10** | **Carro cisterna de la empresa proveedora A** | | | | | | | | | | | |
| **Concentración de CT NMP/100mL** | **<1,1** | **NM** | **NM** | **NM** | **<1,1** | **<1,1** | **NM** | **NM** | **<1,1** | **NM** | **NM** | **NM** |
| **Concentración de CF**  **NMP/100mL** | **<1,1** | **NM** | **NM** | **NM** | **<1,1** | **<1,1** | **NM** | **NM** | **<1,1** | **NM** | **NM** | **NM** |
| **Concentración de *E. coli***  **NMP/100mL** | **<1,1** | **NM** | **NM** | **NM** | **<1,1** | **<1,1** | **NM** | **NM** | **<1,1** | **NM** | **NM** | **NM** |
| **Concentración de Bact.Het**  **UFC/mL (x10^2^)** | **2** | **NM** | **NM** | **NM** | **4,85** | **3,80** | **NM** | **NM** | **5,80** | **NM** | **NM** | **NM** |
| **TLM-CA11** | **Carro cisterna de la empresa proveedora A** | | | | | | | | | | | |
| **Concentración de CT NMP/100mL** | **<1,1** | **NM** | **NM** | **NM** | **5,1** | **<1,1** | **NM** | **NM** | **<1,1** | **NM** | **NM** | **NM** |
| **Concentración de CF**  **NMP/100mL** | **<1,1** | **NM** | **NM** | **NM** | **<1,1** | **<1,1** | **NM** | **NM** | **<1,1** | **NM** | **NM** | **NM** |
| **Concentración de *E. coli***  **NMP/100mL** | **<1,1** | **NM** | **NM** | **NM** | **<1,1** | **<1,1** | **NM** | **NM** | **<1,1** | **NM** | **NM** | **NM** |
| **Concentración de Bact.Het**  **UFC/mL (x10^2^)** | **1,5** | **NM** | **NM** | **NM** | **3,79** | **2,58** | **NM** | **NM** | **6,90** | **NM** | **NM** | **NM** |
| **SAA-TA-01** | **Carro cisterna de la empresa proveedora B** | | | | | | | | | | | |
| **Concentración de CT NMP/100mL** | **NM** | **<1,1** | **NM** | **9,2** | **NM** | **NM** | **<1,1** | **NM** | **NM** | **<1,1** | **NM** | **NM** |
| **Concentración de CF**  **NMP/100mL** | **NM** | **<1,1** | **NM** | **9,2** | **NM** | **NM** | **<1,1** | **NM** | **NM** | **<1,1** | **NM** | **NM** |
| **Concentración de *E. coli***  **NMP/100mL** | **NM** | **<1,1** | **NM** | **<1,1** | **NM** | **NM** | **<1,1** | **NM** | **NM** | **<1,1** | **NM** | **NM** |
| **Concentración de Bact.Het**  **UFC/mL (x10^2^)** | **NM** | **6** | **NM** | **7,3** | **NM** | **NM** | **5,60** | **NM** | **NM** | **0,30** | **NM** | **NM** |
| **SGP-CA-1** | **Carro cisterna de la empresa proveedora C** | | | | | | | | | | | |
| **Concentración de CT NMP/100mL** | **NM** | **NM** | **<1,1** | **NM** | **NM** | **NM** | **NM** | **<1,1** | **NM** | **NM** | **<1,1** | **>23** |
| **Concentración de CF**  **NMP/100mL** | **NM** | **NM** | **<1,1** | **NM** | **NM** | **NM** | **NM** | **<1,1** | **NM** | **NM** | **<1,1** | **<1,1** |
| **Concentración de *E. coli***  **NMP/100mL** | **NM** | **NM** | **<1,1** | **NM** | **NM** | **NM** | **NM** | **<1,1** | **NM** | **NM** | **<1,1** | **<1,1** |
| **Concentración de Bact.Het**  **UFC/mL (x10^2^)** | **NM** | **NM** | **7** | **NM** | **NM** | **NM** | **NM** | **6,80** | **NM** | **NM** | **2,36** | **3,36** |
| **Pozo “lado aire” de empresa proveedora** | **ENE** | **FEB** | **MAR** | **ABR** | **MAY** | **JUN** | **JUL** | **AGO** | **SEP** | **OCT** | **NOV** | **DIC** |
| **PZO-SAA** | **Pozo lado aire de la empresa proveedora B** | | | | | | | | | | | |
| **Concentración de CT NMP/100mL** | **NM** | **<1,1** | **NM** | **<1,1** | **NM** | **NM** | **12** | **NM** | **NM** | **<1,1** | **NM** | **NM** |
| **Concentración de CF**  **NMP/100mL** | **NM** | **<1,1** | **NM** | **<1,1** | **NM** | **NM** | **12** | **NM** | **NM** | **<1,1** | **NM** | **NM** |
| **Concentración de *E. coli***  **NMP/100mL** | **NM** | **<1,1** | **NM** | **<1,1** | **NM** | **NM** | **<1,1** | **NM** | **NM** | **<1,1** | **NM** | **NM** |
| **Concentración de Bact.Het**  **UFC/mL (x10^2^)** | **NM** | **33** | **NM** | **2,88** | **NM** | **NM** | **77,7** | **NM** | **NM** | **0,04** | **NM** | **NM** |
| **Surtidor de agua para consumo humano** | **ENE** | **FEB** | **MAR** | **ABR** | **MAY** | **JUN** | **JUL** | **AGO** | **SEP** | **OCT** | **NOV** | **DIC** |
| **SRT-SWS** | **Surtidor de la empresa proveedora C** | | | | | | | | | | | |
| **Concentración de CT NMP/100mL** | **NM** | **NM** | **<1,1** | **NM** | **NM** | **NM** | **NM** | **<1,1** | **NM** | **NM** | **<1,1** | **>23** |
| **Concentración de CF**  **NMP/100mL** | **NM** | **NM** | **<1,1** | **NM** | **NM** | **NM** | **NM** | **<1,1** | **NM** | **NM** | **<1,1** | **<1,1** |
| **Concentración de *E. coli***  **NMP/100mL** | **NM** | **NM** | **<1,1** | **NM** | **NM** | **NM** | **NM** | **<1,1** | **NM** | **NM** | **<1,1** | **<1,1** |
| **Concentración de Bact.Het**  **UFC/mL (x10^2^)** | **NM** | **NM** | **8** | **NM** | **NM** | **NM** | **NM** | **5,60** | **NM** | **NM** | **0,29** | **0,58** |

**Nota:** Los carros cisternas, pozos y surtidores de las empresas proveedoras de agua para consumo humano A, B y C fueron muestreados trimestralmente (cuatro veces al año). Los meses sin muestreos se consignan como NM.

- **CT:** Coliformes totales
- **CF:** Coliformes fecales o termotolerantes
- **UFC/mL:** Unidades Formadoras de Colonias por mL.
- **<1,1 NMP/100mL:** Resultado por debajo del límite de detección del método.
- **>23 NMP/100mL:** Resultado por encima del límite superior de detección del método.

: Los valores resaltados en rojo indican incumplimiento del Límite Máximo Permisible (LMP) establecido en el Decreto Supremo N° 031-2010-SA, Reglamento de la Calidad del Agua para Consumo Humano del Ministerio de Salud del Perú.

**Métodos empleados:** Coliformes totales, termotolerantes y *E. coli*: Técnica de fermentación de tubos múltiples (NMP), APHA 9221 B, C, E y G ^(15)^. Bacterias heterotróficas: Recuento en placa, APHA 9215 B.

**Material suplementario 4**. Ensayos secuenciales para la detección y confirmación de coliformes por colimetría NMP/100 ml.

**M****aterial suplementario 5**. Aislamiento de *Pseudomonas* spp. a partir de caldo lauril sulfato suplementado con púrpura de bromocresol al 0,01%.

**Material suplementario 6.** Primera parte de la confirmación bioquímica de *Pseudomonas aeruginosa* según la norma ISO 16266:2006.

En la imagen C, se observa la prueba de oxidasa positiva y la prueba de producción de amoniaco a partir del caldo acetamida luego de la siembra e incubación de la cepa de *Pseudomonas aeruginosa* al agregar el Reactivo de Nessler (caldo acetamida: translucido, tubo central y derecha “caldo acetamida + Reactivo de Nessler” positivo a producción de amoniaco: color amarillo a marrón)

**Material suplementario 7.** Segunda parte de la confirmación bioquímica de *Pseudomonas aeruginosa* según la norma ISO 16266:2006.

En la imagen D se observa el crecimiento de la cepa en agar King B, evidenciando fluorescencia positiva bajo luz UV, característica de la producción de pigmentos tipo pioverdina. Asimismo, la cepa presenta prueba de oxidasa positiva y producción de amoníaco a partir de acetamida (prueba de Nessler positiva), evidenciada por el cambio de coloración a amarillo-marrón tras la adición del reactivo.

La concordancia de estos resultados (fluorescencia en agar King B, oxidasa positiva y prueba de acetamida positiva) permite la confirmación bioquímica de Pseudomonas aeruginosa conforme a los criterios establecidos en la norma ISO 16266:2006.

**Material suplementario 8.** Evaluación de la sensibilidad antimicrobiana de aislamientos de *Pseudomonas aeruginosa* (aislamientos SAA-TA3 y SAA-TA4) mediante el método de difusión en disco en agar Mueller-Hinton.
