# Supplementary material for: Microbiological quality and antimicrobial resistance of Pseudomonas aeruginosa in drinking water systems of the old terminal of Jorge Chávez International Airport: potential role as environmental reservoir
Source: Rev Peru Med Exp Salud Publica. 2026 Jun 30;43(2):207–16. doi: 10.17843/rpmesp.2026.432.15160 (PMC13456917; doi:10.17843/rpmesp.2026.432.15160)
Supplement: Translation

# Translation: Calidad microbiológica y resistencia antimicrobiana de *Pseudomonas aeruginosa* en sistemas de agua potable del antiguo terminal del Aeropuerto Internacional Jorge Chávez: potencial papel como reservorio ambiental

**Authors:** Leonardo Enrique Sulca-Galarza, Rigoberto Cilis Robles-Camarena, Tito Libio Sánchez-Rojas, Aldo Franco Rodríguez-Gonzales

**Affiliations:** 1 Dirección de Sanidad Aerea Internacional, Callao, Perú.; 2 Facultad de Ciencias Biologicas, Universidad Nacional Mayor de San Marcos, Lima, Perú.; 3 Direccion Ejecutiva de Sanidades Internacionales, Callao, Perú.; 4 Laboratorio de Biotecnologia Ambiental y Bioprocesos industriales, Facultad de Ciencias Biologicas, Universidad Nacional Mayor de San Marcos, Lima, Perú.

**Keywords:** Pseudomonas aeruginosa, Agua Potable, Farmacorresistencia Microbiana, Aeropuertos, Vigilancia Sanitaria, Calidad del Agua

## Abstract

**Objetivo.:**

Determinar la calidad microbiológica del agua para consumo humano en un aeropuerto internacional del Perú según la normativa nacional, evaluar los patrones fenotípicos de resistencia antimicrobiana de *Pseudomonas aeruginosa* aislada de sistemas hídricos y explorar su posible papel como reservorio ambiental de bacterias resistentes en un punto de entrada internacional.

**Materiales y Métodos.:**

Estudio descriptivo de corte transversal realizado en 2024. Se analizaron 145 muestras de agua procedentes de grifos del terminal aeroportuario, restaurantes, empresas proveedoras, pozos y aeronaves. Los indicadores microbiológicos se evaluaron conforme a la normativa nacional vigente y la susceptibilidad antimicrobiana de 67 aislamientos de *P. aeruginosa* se determinó mediante difusión en disco. Las clasificaciones MDR, XDR y PDR se establecieron según criterios internacionales.

**Resultados.:**

El 50,3% (73/145) de las muestras no cumplió los estándares microbiológicos establecidos por la normativa. *P. aeruginosa* se detectó en el 55,8% (81/145) de las muestras. De los 67 aislamientos analizados, el 38,8% fue clasificado como MDR y el 11,9% como XDR; no se identificaron aislamientos PDR. Se observaron concentraciones superiores a 16 NMP/100 mL en múltiples puntos del sistema, incluidas aeronaves.

**Conclusiones.:**

La detección de *P. aeruginosa* resistente en sistemas hídricos aeroportuarios sugiere vulnerabilidades sanitarias y un posible papel como reservorio ambiental de bacterias resistentes. Se recomienda fortalecer la vigilancia microbiológica e incorporar la evaluación de la resistencia antimicrobiana en los programas de monitoreo de la calidad del agua en puntos de entrada internacionales.

## INTRODUCCIÓN

El Aeropuerto Internacional Jorge Chávez en el Callao (AIC), cuyas operaciones fueron trasladadas a una nueva terminal de pasajeros, constituye el principal punto de entrada aérea internacional del Perú y concentra un elevado flujo de pasajeros, aeronaves y actividades operativas asociadas ^1^. Estas características lo convierten en un entorno de interés para la vigilancia sanitaria y para la evaluación de la calidad microbiológica del agua destinada al consumo humano en sistemas complejos de distribución y abastecimiento.

En ese sentido, el riesgo para la salud pública se determina por la gravedad y desarrollo de enfermedades asociadas a agentes patógenos, su infectividad y la población expuesta ^2^. Sin embargo, en las poblaciones vulnerables como lactantes, niños de corta edad, personas debilitadas inmunológicamente o inmunodeprimidos y ancianos ^3^, su principal forma de transmisión es el consumo de agua contaminada; pero existen otras vías como el contacto entre personas, la ingesta de alimentos, gotitas y aerosoles ^2^. Por ello, para garantizar la calidad del agua de consumo humano y proteger la salud pública en los aeropuertos, es fundamental implementar marcos de inocuidad y planes integrales de seguridad del suministro, con el fin de prevenir brotes de enfermedades transmitidas por el agua que podrían afectar a numerosos viajeros ^2^.

La resistencia bacteriana se encuentra como una de las amenazas para la salud pública mundial, con millones de muertes atribuibles anualmente y proyecciones crecientes hacia 2050 ^4^. La distribución, prevalencia y diversidad de bacterias portadoras de resistencia a los antimicrobianos presentan marcadas diferencias entre distintas regiones del mundo, debido a la influencia de factores sanitarios, condiciones socioeconómicas, genéticas y variables ambientales ^4^^,^^5^. Asimismo, esta heterogeneidad se intensifica en el contexto de la globalización, ya que el aumento de los desplazamientos internacionales y del comercio transfronterizo facilita el intercambio o transferencia horizontal de genes y la diseminación global de bacterias resistentes ^6^. Es probable que pasajeros infectados con patógenos los eliminen en sus heces y fluidos corporales (orina, saliva, esputo, etc.) al hacer uso de los servicios higiénicos del aeropuerto y de las aeronaves ^7^.

Según el DS 031-2010-SA ^8^, el principal indicador microbiológico de la calidad del agua para consumo humano son los coliformes, especialmente los coliformes termotolerantes y *Escherichia coli*, como evidencia de contaminación fecal reciente ^9^. La norma establece límites de <1,1 NMP/100 mL o <1 UFC/100 mL; sin embargo, un solo indicador no garantiza la inocuidad frente a la diversidad de patógenos potenciales. En este contexto, *Pseudomonas aeruginosa* no es considerada en el control rutinario de agua potable, aunque sí figura como criterio microbiológico en bebidas envasadas ^10^ y en la normativa sanitaria vigente para piscinas de uso colectivo ^11^, evidenciándose cierta falta o sesgo en la normativa peruana sobre la calidad del agua de consumo humano, que excluye en el control microbiológico a *Pseudomonas aeruginosa*, un bacilo gramnegativo conocido por su resistencia a múltiples antibióticos, patógeno oportunista que puede contaminar el agua y representar un riesgo significativo para la salud humana, clasificado como prioridad alta por su resistencia a carbapenémicos ^12^. Su capacidad para sobrevivir en ambientes adversos y desarrollar resistencia a agentes antimicrobianos lo convierte en un indicador relevante para evaluar la calidad microbiológica del agua, además de su capacidad de inhibir los coliformes ^13^. Por otro lado, la ausencia de bacterias coliformes en las muestras de agua de cisterna y tanques no significa la ausencia de riesgo microbiológico, pudiéndose encontrar *Pseudomonas aeruginosa* por su metabolismo versátil, resistencia y como patógeno oportunista ^14^.

En 2019, las *Pseudomonas aeruginosa* generaron aproximadamente 84600 muertes atribuibles a resistencia antimicrobiana a uno o más antibióticos ^4^. Al analizar las combinaciones patógeno-fármaco, la mayor carga de mortalidad se observó en la resistencia a carbapenémicos (≈38 100 muertes), seguida de fluoroquinolonas (≈18 300 muertes) y antibióticos con actividad antipseudomónica (≈10 300 muertes). También se registraron muertes atribuibles a resistencia frente a cefalosporinas de tercera generación (≈10 400), cefalosporinas de cuarta generación (≈4370) y aminoglucósidos (≈3010). Estos hallazgos evidencian que, aunque *Pseudomonas aeruginosa* mantiene una elevada carga de mortalidad asociada a resistencia, el mayor impacto clínico se concentra en la pérdida de eficacia de los carbapenémicos, considerados fármacos de última línea en infecciones graves ^4^.

El presente estudio evalúa la calidad del agua para consumo humano en distintos puntos del AIC, con énfasis en la detección y resistencia antimicrobiana de *Pseudomonas aeruginosa*, bacteria considerada por la Organización Mundial de la Salud (OMS) como un patógeno prioritario ^12^. En este contexto, se busca generar evidencia actualizada sobre la contaminación del agua y la presencia de cepas resistentes, con el fin de identificar riesgos sanitarios en un punto de entrada internacional.

**MENSAJES CLAVE**

Motivación del estudio. Existe limitada evidencia sobre la presencia de *Pseudomonas aeruginosa* y su resistencia antimicrobiana en sistemas de agua potable en aeropuertos en América Latina, pese a su relevancia como puntos de alta conectividad internacional.

Principales hallazgos. Se identificó un porcentaje considerable de muestras que no cumplían con los estándares microbiológicos de la normativa nacional e internacional, así como una frecuencia significativa de cepas de *P. aeruginosa* con resistencia antimicrobiana, incluyendo fenotipos multidrogoresistentes y extremadamente resistentes.

Implicancias en salud pública. Los resultados sugieren que los sistemas hídricos aeroportuarios podrían actuar como potenciales reservorios ambientales de bacterias resistentes, lo que respalda la necesidad de fortalecer la vigilancia microbiológica y la actualización de la normativa sanitaria.

## MATERIALES Y MÉTODOS

### Diseño y área del estudio

Se realizó un estudio observacional descriptivo de corte transversal en el AIC, ubicado en la Provincia Constitucional del Callao. El AIC es considerado uno de los tres aeropuertos más importantes de Sudamérica y se encuentra bajo la jurisdicción de la Dirección Regional de Salud (DIRESA) Callao. Opera las 24 horas del día y presta servicios a aerolíneas comerciales nacionales e internacionales, contando con 24 aerolíneas internacionales y 5 nacionales. En 2023, el aeropuerto registró aproximadamente 21,2 millones de pasajeros, lo que representó un incremento del 14,1% respecto al año anterior; se espera que esta cifra aumente significativamente con la entrada en funcionamiento del nuevo terminal aeroportuario ^1^.

### Puntos de muestreo

La selección de puntos de muestreo se realizó bajo un enfoque de riesgo sanitario, priorizando puntos críticos de transferencia y consumo definidos en la Guía de Higiene y Saneamiento en el Transporte Aéreo y en el marco del Plan de Inocuidad del Agua para aeropuertos; se consideraron 16 unidades de muestreo: 9 puntos fijos, 6 puntos variables codificados y una categoría adicional correspondiente a aeronaves comerciales. Los puntos fijos incluyeron grifos de baños de acceso público, grifos de restaurantes, el grifo de la cocina de la Dirección de Sanidad Aérea Internacional y un pozo de agua para consumo humano que abastece a la terminal aeroportuaria. Los puntos variables codificados correspondieron a carros cisterna proveedores de agua para aeronaves, un surtidor de agua para consumo humano y un pozo asociado a dicho sistema. Adicionalmente, se incluyeron muestras procedentes de aeronaves comerciales, las cuales fueron consideradas una unidad de muestreo variable debido a que las aeronaves evaluadas variaron durante el periodo de estudio.

Los puntos fijos fueron evaluados mensualmente durante todo el periodo de estudio, generándose mediciones repetidas en los mismos puntos de muestreo. Los puntos variables fueron evaluados según disponibilidad operativa y condiciones del sistema de abastecimiento. En el caso de las empresas proveedoras de agua para aeronaves, se planificó un muestreo rotatorio trimestral entre las empresas; sin embargo, por limitaciones operativas, no fue posible mantener estrictamente dicha rotación. No obstante, se realizaron 4 evaluaciones por empresa durante el año, obteniéndose 2 muestras por visita, alcanzando un total de 8 muestras por empresa.

Respecto a las aeronaves, las muestras fueron obtenidas de unidades distintas en cada ocasión, correspondientes a 5 vuelos internacionales y 8 nacionales, por lo que estas observaciones se consideran independientes entre sí.

### Número de muestras y tiempo de muestreo

El total de muestras mensuales recolectadas fueron alrededor de 12, entre enero y diciembre del 2024 dando un total de 145 muestras (en diciembre se tomaron 13 muestras). Estas muestras incluyeron tanto puntos fijos evaluados de forma repetida como puntos variables evaluados de manera no sistemática a lo largo del periodo de estudio.

La recolección, transporte y conservación de las muestras de agua se realizó por personal profesional y técnicos de salud ambiental de la Dirección de Sanidad Aérea Internacional, siguiendo las recomendaciones del American Public Health Association, American Water Works Association y Water Environment Federation (APHA, AWWA y WEF) ^15^.

### Calidad microbiológica y metodología

Las evaluaciones microbiológicas se realizaron sobre cada muestra individual recolectada, siguiendo los procedimientos estandarizados para la evaluación de la calidad microbiológica del agua para consumo humano. Se utilizaron los indicadores bacterianos recomendados por la normativa DS 031-2010-SA Reglamento de la calidad del agua potable para consumo humano ^8^: coliformes totales, coliformes termotolerantes, *Escherichia coli* y bacterias heterotróficas; adicionalmente, se incluyó *Pseudomonas aeruginosa* como indicador complementario de riesgo sanitario de acuerdo a la Guía de Higiene y Saneamiento de los Transportes Aéreos y Guías para la calidad del agua de consumo humano de la OMS.

La metodología utilizada para la determinación de la calidad microbiológica del agua potable fue la técnica de fermentación en tubos múltiples por número más probable (NMP/100 mL), conforme a los métodos estándar de APHA, AWWA, WEF ^15^. El límite de detección del método fue <1,1 NMP/100 mL, mientras que el límite superior fue de >23 NMP/100 mL. Para *Pseudomonas aeruginosa* se modificó el método de Ramírez Z. ^9^, Álvarez Guevara J. *et al.*^16^ y se siguieron los criterios de confirmación de acuerdo con la ISO 16266:2006 ^17^. Su límite de detección del método fue <2,2 NMP/100 mL, mientras que el límite superior fue de >16 NMP/100 mL.

Para la cuantificación de las bacterias heterotróficas se utilizó agar Plate Count por vertido de acuerdo con el método 9215 Recuento de placas heterotróficas del APHA AWWA. El límite de detección fue de 1 UFC/mL y el rango contable fue de 30-300 UFC/mL.

Para la determinación de la resistencia fenotípica de *Pseudomonas aeruginosa* a antibióticos, se utilizó el método de difusión en disco, de acuerdo con la metodología descrita por el Instituto Nacional de Salud (INS)-MINSA del 2002 ^18^ y por el Instituto de Estándares Clínicos y de Laboratorios ^19^. Los antibióticos probados fueron: ceftazidima (CAZ-30µg), cefepime (FEP-30µg), gentamicina (GM-10µg), amikacina (AMK-30µg), imipenem (IPM-10µg), meropenem (MEM-10µg), cefoperazona-sulbactam (SUL-30µg), aztreonam (AZT-30µg), ciprofloxacina (CIP-5µg) y norfloxacina (NX-10µg), los cuales fueron adquiridos comercialmente. Para la determinación de los aislamientos resistentes se siguió la terminología propuesta por Magiorakos A. *et al.*^20^ en relación con la definición de las diferentes bacterias resistentes a múltiples fármacos (MDR), extremadamente resistentes (XDR) y panresistentes (PDR).

Se implementaron controles de calidad en todas las etapas de los análisis microbiológicos. Como controles de medio, reactivo y controles positivos se utilizó la cepa de referencia *Pseudomonas aeruginosa* ATCC 27853 y *E. coli* ATCC 25992; como controles negativos se utilizaron cepas de *Staphylococcus aureus* ATCC 6538, *Enterobacter aerogenes* ATCC 13048 y *Enterobacter cloacae* ATCC 13047 para la técnica de fermentación de tubos múltiples.

*Numeración de coliformes y Escherichia coli*

El aislamiento primario se realizó utilizando caldo lauril sulfato doble concentración suplementado con púrpura de bromocresol al 0,01%, inoculados e incubados en serie de 10 tubos durante 24-48 horas a 35 °C. Posteriormente se observó el cambio de color del medio de púrpura a amarillo por la acidificación del medio; con sospecha de coliformes se prosiguió con la inoculación en caldo bilis verde brillante por 48 horas a 35 °C, para confirmar coliformes totales, y caldo EC y agua triptona por 24 horas a 44,5 °C, para coliformes termotolerantes y presencia de *Escherichia coli*, respectivamente. La prueba de indol se hizo con reactivo de Kovács a partir del agua triptona ^15^.

*Numeración de Pseudomonas aeruginosa*

La detección de *Pseudomonas aeruginosa* se realizó mediante una adaptación del método convencional, integrando su aislamiento a partir de los tubos de caldo lauril sulfato suplementados con púrpura de bromocresol al 0,01% utilizados en la técnica de NMP para coliformes. Se seleccionaron aquellos tubos con crecimiento, turbidez o formación de película superficial sin cambio de coloración, los cuales fueron incubados adicionalmente durante 24 h a 36 °C. Posteriormente, se sembraban en agar cetrimide y se incubaron hasta 48 h a 36 °C. Las colonias presuntivas fueron subcultivadas en agar King B y se incubaron 3-5 días a 35 °C, evaluando la producción de fluorescencia con lámpara ultravioleta de onda larga (365 nm). Las colonias presuntivas de *Pseudomonas aeruginosa* se confirmaron mediante producción de amoníaco a partir de acetamida (reactivo de Nessler) y prueba de oxidasa positiva. Las colonias positivas en estas tres pruebas se consideraron *Pseudomonas aeruginosa*, conforme con la norma 16266:2006 ^17^.

*Cuantificación de bacterias heterotróficas*

Se siguió el método 9215 Recuento de placas heterotróficas del APHA, AWWA y WEF ^15^, donde a partir de las muestras se realizaron diluciones seriadas y se sembraron 0,1 mL por duplicado por cada dilución, en placas Petri con agar Plate Count, y se incubaron por 48 horas a 35 °C ^15^.

### Análisis estadístico

La unidad de análisis fue la muestra de agua individual (n=145), sobre la cual se calcularon las frecuencias absolutas, porcentajes y distribuciones de los indicadores microbiológicos evaluados. Las muestras fueron recolectadas de 16 puntos de muestreo, incluyendo 9 puntos fijos evaluados de manera mensual durante todo el periodo de estudio, lo que generó mediciones repetidas en los mismos puntos y, por tanto, una potencial dependencia entre observaciones. En contraste, las muestras provenientes de aeronaves correspondieron a unidades distintas en cada ocasión, considerándose independientes entre sí.

Dado que el estudio tuvo un enfoque descriptivo, los resultados se presentan a nivel de muestra, sin realizar análisis inferenciales ni modelar la correlación intra-punto. El procesamiento de datos se realizó utilizando Microsoft Excel.

### Aspectos éticos

El presente estudio se desarrolló en el marco de las competencias de vigilancia sanitaria de la Dirección de Sanidad Aérea Internacional, órgano adscrito a la Dirección Ejecutiva de Sanidades Internacionales de la Dirección Regional de Salud del Callao, y cuenta con la aprobación del Comité de Ética institucional correspondiente (Oficios: Constancia N.° 001-2024 y N.° 041-2024-Comité de Ética/UI/DIRESA/Callao).

## RESULTADOS

Se analizaron 145 muestras de agua potable provenientes de grifos del terminal aeroportuario, restaurantes, empresas proveedoras de agua, pozos y aeronaves durante el año 2024. La clasificación de muestras como aptas o no aptas se realizó exclusivamente en función de los parámetros microbiológicos establecidos en el DS N.° 031-2010-SA, sin considerar la presencia de *Pseudomonas aeruginosa*, la cual fue evaluada como indicador complementario. El 50,3% (73/145) fueron clasificadas como no aptas para consumo humano (tabla 1). En relación con los indicadores microbiológicos evaluados, la presencia de coliformes totales se detectó en el 12,4% (18/145) de las muestras, coliformes termotolerantes en el 9,7% (14/145) y *E. coli* en el 2,1% (3/145). Las bacterias heterotróficas superaron el límite máximo permisible en el 45,5% de las muestras (66/145). Asimismo, *P. aeruginosa* fue aislada en el 55,9% (81/145) del total analizado.

**Tabla 1:** Frecuencia de incumplimiento de los parámetros microbiológicos establecidos para agua de consumo humano en 145 muestras analizadas durante el 2024.

Parámetro microbiológico	Muestras que superan el límite máximo permisible (n/N)	%
Coliformes totales	18/145	12,4
Coliformes termotolerantes	14/145	9,7
*Escherichia coli*	3/145	2,1
Bacterias heterotróficas	66/145	45,5
Muestras no aptas^a^	73/145	50,3

a Muestras que incumplieron al menos uno de los parámetros microbiológicos establecidos en el DS N.° 031-2010-SA.

Los valores correspondientes a cada parámetro no son mutuamente excluyentes, por lo que su suma puede ser mayor que el total de muestras no aptas.

Métodos empleados: coliformes totales, termotolerantes y *E. coli*: técnica de fermentación de tubos múltiples (NMP), APHA, AWWA y WEF 9221 B, C, E y G ^15^. Bacterias heterotróficas: recuento en placa, APHA, AWWA y WEF 9215 B.

En las aeronaves de vuelos nacionales e internacionales se observaron detecciones frecuentes de *P. aeruginosa*, con concentraciones >16 NMP/100 mL en varios meses. Los recuentos de bacterias heterotróficas oscilaron entre 34 y 2560 UFC/mL, con valores más elevados durante el tercer trimestre. Además, se registraron episodios aislados de coliformes >23 NMP/100 mL y detección puntual de *E. coli* (tabla 2). Al analizar la distribución de la contaminación microbiológica según tipo de punto de muestreo, se identificaron diferencias entre los componentes del sistema hídrico. En los grifos del terminal aeroportuario se observaron incrementos episódicos de coliformes, con valores máximos de hasta 9,2 NMP/100 mL y detecciones puntuales de *E. coli.* En restaurantes se registraron episodios transitorios con concentraciones >23 NMP/100 mL de coliformes totales y termotolerantes. En pozos, surtidores y camiones cisterna predominaron valores bajos durante la mayor parte del periodo de estudio, aunque se observaron incrementos ocasionales >23 NMP/100 mL. Respecto a *P. aeruginosa*, los grifos del terminal presentaron concentraciones >16 NMP/100 mL en varios meses, mientras que en pozos y empresas proveedoras las concentraciones fueron generalmente ≤2,2 NMP/100 mL, con incrementos transitorios que alcanzaron 16 y >16 NMP/100 mL (tabla 3). Para la evaluación fenotípica de resistencia antimicrobiana se analizaron 67 aislamientos de *P. aeruginosa*. El 38,8% (26/67) fue clasificado como multidrogoresistente (MDR), el 11,9% (8/67) como extensamente resistente (XDR) y no se identificaron aislamientos panresistentes (PDR). El 49,3% restante presentó resistencia extendida (tabla 4). Se observó variabilidad en los perfiles de susceptibilidad, con resistencia predominante frente a aminoglucósidos y algunas cefalosporinas, mientras que la mayoría de los aislamientos conservó sensibilidad frente a carbapenémicos (figura 1).

**Tabla 2:** Resultados microbiológicos individuales de 13 muestras de agua para consumo humano obtenidas de aeronaves comerciales de vuelos nacionales e internacionales durante el 2024.

Aerolínea	Mes	Coliformes totales NMP/100mL	Coliformes termotolerantes NMP/100mL	***E. coli* NMP/100mL**	Bacterias heterotróficas UFC/mL	***P. aeruginosa* NMP/100mL**
A1 Internacional	Enero	<1,1	<1,1	<1,1	133	>16
A2 Nacional	Febrero	>23	>23	<1,1	675	>16
A1 Nacional	Marzo	<1,1	<1,1	<1,1	67	>16
A3 Nacional	Abril	<1,1	<1,1	<1,1	95	>16
A1 Nacional	Mayo	<1,1	<1,1	<1,1	112	>16
A1 Internacional	Junio	<1,1	<1,1	<1,1	98	>16
A1 Nacional	Julio	<1,1	<1,1	<1,1	34	<2,2
A3 Nacional	Agosto	>23	>23	12	2560	>16
A3 Internacional	Setiembre	>23	16	<1,1	835	>16
A1 Nacional	Octubre	<1,1	<1,1	<1,1	51	5,1
A3 Nacional	Noviembre	<1,1	<1,1	<1,1	420	2,2
A3 Internacional	Diciembre	3,6	3,6	<1,1	730	>16
A1 Internacional	Diciembre	1,1	1,1	<1,1	610	>16

Las 13 muestras presentadas corresponden a las muestras tomadas en los grifos de las aeronaves comerciales dentro de las 145 muestras de agua para consumo humano analizadas durante el periodo de estudio. Cada fila corresponde a una muestra independiente obtenida de una aeronave comercial (8 vuelos nacionales y 5 internacionales).

Los valores expresados como “<” y “>” corresponden a los límites de cuantificación del método de Número Más Probable (NMP) utilizado para la determinación de coliformes y *P. aeruginosa*.

Las aerolíneas fueron codificadas (A1, A2 y A3) para preservar la confidencialidad de la información.

**Tabla 3:** Resultados mensuales de *Pseudomonas aeruginosa* en muestras de agua para consumo humano obtenidas en instalaciones aeroportuarias y sistemas asociados de abastecimiento durante el 2024.

Código de muestra		**Meses de muestreo y resultados para *P. aeruginosa* (NMP/100mL)**											
		En.	Feb.	Mar.	Abr.	May.	Jun.	Jul.	Ag.	Set.	Oct.	Nov.	Dic.
Grifos de la terminal aeroportuaria													
	GT-01 - Comedor de la instalación administrativa interna (1er nivel)	<2,2	2,2	<2,2	<2,2	9,2	>16	>16	5,1	2,2	>16	<2,2	>16
	GT-02 - Baño ubicado al lado de la instalación administrativa (1er nivel)	<2,2	9,2	<2,2	<2,2	<2,2	<2,2	<2,2	<2,2	2,2	<2,2	2,2	>16
	GT-03 - Baño al lado del patio de comidas zona nacional (2do nivel)	5,1	9,2	<2,2	<2,2	<2,2	>16	>16	<2,2	9,2	<2,2	<2,2	>16
	GT-04 - Baño frente al área de objetos perdidos (1er nivel)	2,2	9,2	<2,2	<2,2	<2,2	>16	>16	<2,2	<2,2	<2,2	2,2	2,2
Pozo de la terminal aeroportuaria													
	PZ-01 - Grifo del 1er baño cercano al pozo N.° 1	2,2	2,2	<2,2	<2,2	<2,2	2,2	2,2	2,2	<2,2	<2,2	2,2	2,2
Grifos de restaurantes													
	GR-01 - Grifo de cocina zona nacional (2do nivel)	>16	<2,2	<2,2	<2,2	<2,2	>16	2,2	>16	<2,2	<2,2	<2,2	<2,2
	GR-02 - Grifo de cocina zona nacional (2do nivel)	16	<2,2	<2,2	<2,2	2,2	>16	2,2	>16	<2,2	<2,2	<2,2	<2,2
	GR-03 - Grifo del bar zona internacional (2do nivel)	<2,2	<2,2	<2,2	<2,2	2,2	2,2	>16	2,2	>16	<2,2	>16	>16
	GR-04 - Grifo de cocina zona internacional (2do nivel)	5,1	<2,2	<2,2	5,1	5,1	5,1	>16	5,1	>16	>16	>16	>16
Carros cisterna de las empresas proveedoras													
	CI-01 - Carro cisterna de la empresa proveedora A	<2,2	NM	NM	NM	16	<2,2	NM	NM	2,2	NM	NM	NM
	CI-02 - Carro cisterna de la empresa proveedora A	5,1	NM	NM	NM	>16	<2,2	NM	NM	2,2	NM	NM	NM
	CI-03 - Carro cisterna de la empresa proveedora B	NM	<2,2	NM	16	NM	NM	<2,2	NM	NM	<2,2	NM	NM
	CI-04 - Carro cisterna de la empresa proveedora C	NM	NM	<2,2	NM	NM	NM	NM	9,2	NM	NM	<2,2	>16
Pozo “lado aire” de empresa proveedora													
	PZ-02 - Pozo lado aire de la empresa proveedora B	NM	2,2	NM	<2,2	NM	NM	>16	NM	NM	<2,2	NM	NM
Surtidor de agua para consumo humano													
	SR-01 - Surtidor de la empresa proveedora C	NM	NM	<2,2	NM	NM	NM	NM	9,2	NM	NM	<2,2	>16

NM: no muestreado. <2,2 NMP/100 mL: resultado por debajo del límite de detección del método. >16 NMP/100 mL: resultado por encima del límite superior de detección del método.

La tabla presenta resultados individuales obtenidos en los puntos de muestreo evaluados durante el 2024.

Los puntos fijos fueron muestreados mensualmente, mientras que los puntos asociados a empresas proveedoras fueron evaluados según el plan de muestreo establecido.

Los muestreos para cada empresa proveedora de agua para consumo humano A, B y C se tomaron 4 veces al año.

Métodos empleados: se empleó el método de Número Más Probable (NMP) modificado de Ramírez Z. ^9^, Guevara J. et al. ^16^, y se siguieron los criterios de confirmación de acuerdo con la ISO 16266 ^17^.

**Tabla 4:** Distribución de aislamientos de *Pseudomonas aeruginosa* evaluados para susceptibilidad antimicrobiana según perfil de resistencia (MDR, XDR y No MDR/XDR) y puntos de muestreo (n=67).

Puntos de muestreo / Código de laboratorio			Aislamientos evaluados (n)	MDR (n)	XDR (n)	No MDR/XDR (n)
Grifos de la terminal aeroportuaria						
		GT-01	5	2	2	1
		GT-02	1	1	0	0
		GT-03	4	1	0	3
		GT-04	4	2	0	2
Pozos						
		PZ-01	5	2	1	2
		PZ-02	7	4	1	2
Carros cisterna						
		CI-01	1	0	0	1
		CI-02	1	0	0	1
		CI-03	5	1	0	4
		CI-04	2	2	0	0
Surtidor						
		SR-01	2	1	0	1
Grifos de restaurantes						
		GR-01	3	2	0	1
		GR-02	3	2	0	1
		GR-03	6	1	2	3
		GR-04	8	3	2	3
Vuelos comerciales						
	Vuelos nacionales		6	0	0	6
		AE-N01	1	0	0	1
		AE-N02	3	0	0	3
		AE-N03	1	0	0	1
		AE-N04	1	0	0	1
	Vuelos internacionales		4	2	0	2
		AE-I01	2	0	0	2
		AE-I02	1	1	0	0
		AE-I03	1	1	0	0
Total, n (%)			67 (100)	26 (38,8)	8 (11,9)	33 (49,3)

MDR: multidrogoresistente. XDR: extremadamente resistente. No MDR/XDR: aislamientos que no cumplen los criterios de multidrogoresistencia ni resistencia extrema.

El número de aislamientos incluidos en las pruebas de susceptibilidad antimicrobiana no corresponde necesariamente al número de meses positivos registrados por cada punto de muestreo. Los aislamientos fueron obtenidos durante el periodo de estudio, conservados y posteriormente reactivados para su procesamiento conjunto mediante antibiograma. En consecuencia, un punto de muestreo pudo aportar más de un aislamiento o un número menor de aislamientos que el total de aislamientos positivos registrados.

**Figura 1.** Porcentajes de resistencia y sensibilidad a antibióticos in vitro de *Pseudomonas aeruginosa* aisladas del agua para consumo humano del antiguo terminal del Aeropuerto Internacional Jorge Chávez, 2024.

MEM: meropenem, IPM: imipenem, FEP: cefepime, CAZ: ceftazidima, GM: gentamicina, AMK: amikacina, AZT: aztreonam, CIP: ciprofloxacina, NX: norfloxacina, SUL: cefoperazona-sulbactam.

## DISCUSIÓN

El transporte aéreo internacional facilita la diseminación global de bacterias resistentes y otros patógenos entre regiones geográficas ^21^^,^^22^. En un estudio realizado por Handschuh H. et al. (2017), se demostró que las gammaproteobacterias predominantes en el agua de baños y despensas de aeronaves pertenecen al género *Pseudomonas*, actuando como reservorios potenciales de resistencia antimicrobiana ^21^. Asimismo, se identificó la presencia de diversos patógenos oportunistas, entre ellos *P. aeruginosa*, con capacidad de colonizar sistemas hídricos de aeronaves ^23^^,^^24^. En este contexto, la identificación de cepas multidrogoresistentes (MDR) y extremadamente resistentes (XDR) en sistemas de abastecimiento aeroportuarios sugiere que estas infraestructuras podrían funcionar como reservorios ambientales, con potencial de propagación transfronteriza ^25^.

El 55,2% de los aislamientos mostró resistencia a al menos un antimicrobiano, con mayores niveles frente a ceftazidima (56,7%) y aztreonam (53,7%), seguidos de cefepime (34,3%). La resistencia a carbapenémicos como meropenem (44,8%) e imipenem (20,9%) es particularmente relevante debido a su asociación con alta morbimortalidad y limitación terapéutica ^14^^,^^25^. La resistencia observada frente a aminoglucósidos y quinolonas, así como a cefoperazona-sulbactam, sugiere la participación de múltiples mecanismos intrínsecos y adquiridos descritos en *P. aeruginosa*, tales como la producción de betalactamasas, la sobreexpresión de bombas de eflujo y la disminución de la permeabilidad de la membrana externa ^24^. Estos hallazgos coinciden con reportes internacionales sobre *P. aeruginosa* resistente en agua potable y en infraestructuras críticas, aunque la evidencia específica en aeropuertos sigue siendo limitada ^25^. Asimismo, los indicadores coliformes y bacterias heterotróficas evidencian vulnerabilidades del sistema hídrico y condiciones que podrían favorecer la formación de biopelículas y persistencia de *P. aeruginosa* en la red de distribución.

En este contexto, es importante interpretar con cautela la proporción de muestras clasificadas como “no aptas” (50,3%), dado que esta refleja el incumplimiento de criterios microbiológicos normativos en un conjunto de puntos heterogéneos del sistema hídrico, evaluados bajo un enfoque de muestreo basado en riesgo. Al considerar la estratificación por tipo de punto de muestreo, se evidencian patrones diferenciados, donde los sistemas de distribución internos y entornos de uso intensivo (como grifos del terminal, restaurantes y aeronaves) presentan mayor variabilidad y episodios de contaminación, en contraste con fuentes como pozos y empresas proveedoras, que mostraron mayor estabilidad microbiológica. En este sentido, los hallazgos sugieren la coexistencia de riesgos sanitarios asociados al incumplimiento normativo y fenómenos de contaminación operacional vinculados a la dinámica propia de sistemas complejos de distribución de agua en infraestructuras aeroportuarias.

Si bien *P. aeruginosa* no forma parte de los criterios microbiológicos básicos de cumplimiento para la evaluación rutinaria de la potabilidad del agua según la normativa nacional vigente, su presencia es considerada en las guías de calidad de agua para consumo humano de la OMS como un indicador relevante en contextos específicos, particularmente en sistemas de distribución donde puede evidenciar formación de biopelículas, colonización ambiental y posible contaminación operacional, especialmente en redes internas y sistemas de distribución complejos como los aeroportuarios. En este sentido, la elevada frecuencia observada podría estar relacionada con la capacidad de este microorganismo para sobrevivir en ambientes con baja disponibilidad de nutrientes. Su presencia puede representar un riesgo potencial para la población susceptible. No obstante, su relevancia radica en su carácter de patógeno oportunista, especialmente en poblaciones vulnerables, así como en su papel como reservorio de resistencia antimicrobiana, lo que justifica su inclusión como indicador complementario en contextos de vigilancia sanitaria en infraestructuras de alta complejidad como aeropuertos.

Investigaciones en aeropuertos europeos y asiáticos han documentado hallazgos similares, vinculando la contaminación a redes de distribución internas y vehículos de abastecimiento ^26^^-^^29^. Aunque no existe evidencia concluyente de transmisión directa de infecciones por consumo de agua en aeronaves ^23^, la subnotificación puede estar influenciada por el periodo de incubación y la alta movilidad de pasajeros ^27^.

En América Latina, estudios locales han reportado *P. aeruginosa* resistente en fuentes de agua, aunque con menor frecuencia de resistencia que la observada en este estudio ^16^^,^^28^. Esto subraya la importancia de la vigilancia local, dado que los patrones de resistencia varían según contexto geográfico. A nivel global, *P. aeruginosa* resistente a carbapenémicos constituye una de las principales amenazas sanitarias, ubicándose entre los patógenos con mayor carga atribuible de mortalidad ^4^. Modelos de riesgo han demostrado que la colonización intestinal tras la ingesta de agua contaminada es baja en condiciones habituales, pero aumenta significativamente ante altas concentraciones bacterianas y uso concomitante de antibióticos ^30^. En individuos inmunocomprometidos, este riesgo puede ser clínicamente relevante. La persistencia de *P. aeruginosa* en agua potable se asocia a su capacidad de formar biopelículas, tolerar cloro residual y adaptarse a entornos con bajo contenido nutricional ^29^, lo que explica su supervivencia en redes de distribución. La detección de cepas MDR y XDR en un aeropuerto internacional, entorno de alta conectividad, amplía la discusión más allá de la vigilancia tradicional de enfermedades transmisibles, incorporando la resistencia antimicrobiana como componente crítico de bioseguridad en estas zonas.

Entre las limitaciones del estudio se incluyen el acceso restringido a ciertos puntos críticos del sistema hídrico y el enfoque de muestreo basado en riesgo sanitario. La selección de los puntos de muestreo fue de carácter no probabilístico y priorizó componentes considerados críticos para la vigilancia sanitaria, por lo que los resultados no pueden extrapolarse directamente a la totalidad de la red de distribución. Adicionalmente, el número de muestras obtenidas no fue uniforme entre los distintos puntos evaluados debido a diferencias operativas y a la naturaleza de los componentes del sistema, generando una distribución asimétrica de las observaciones. Asimismo, la presencia de mediciones repetidas en puntos fijos a lo largo del tiempo introduce dependencia entre observaciones, la cual no fue modelada estadísticamente debido al enfoque descriptivo del estudio, por lo que los resultados deben interpretarse a nivel de muestra y no como observaciones completamente independientes. La ausencia de caracterización molecular de los aislamientos constituye una limitación relevante, ya que no permite determinar si las detecciones recurrentes de *P. aeruginosa* corresponden a la persistencia de un mismo clon bacteriano dentro del sistema hídrico o a eventos independientes de contaminación. En consecuencia, aunque los hallazgos sugieren condiciones favorables para la persistencia de este microorganismo, no permiten confirmar mecanismos específicos de mantenimiento o dispersión dentro de la red. Asimismo, no se realizaron evaluaciones directas de formación de biopelículas, consideradas uno de los principales mecanismos de persistencia de *P. aeruginosa* en sistemas de distribución de agua, por lo que su participación en los hallazgos observados no pudo ser demostrada. Finalmente, el estudio fue realizado en la infraestructura del antiguo terminal del aeropuerto, por lo que los resultados no pueden extrapolarse directamente a la nueva infraestructura aeroportuaria. A pesar de estas limitaciones, los hallazgos respaldan la necesidad de fortalecer la vigilancia microbiológica en puntos de entrada internacionales e implementar estrategias preventivas para la gestión de la calidad del agua, en concordancia con los principios del Reglamento Sanitario Internacional y con regulaciones específicas aplicables a sistemas de agua en aeronaves ^31^^,^^32^. Asimismo, enfoques emergentes de vigilancia ambiental, incluido el monitoreo de aguas residuales en aeronaves y aeropuertos, han demostrado utilidad para la detección temprana de patógenos y podrían complementar los sistemas tradicionales de vigilancia sanitaria ^33^^,^^34^.

En conclusión, los resultados evidenciaron una elevada frecuencia de incumplimiento de los parámetros microbiológicos establecidos para agua de consumo humano y una presencia recurrente de *P. aeruginosa* con perfiles de resistencia antimicrobiana en distintos componentes del sistema hídrico aeroportuario, incluidas aeronaves. Estos hallazgos sugieren la existencia de condiciones que podrían favorecer la persistencia y circulación de bacterias resistentes en determinados puntos del sistema. Asimismo, constituyen una línea base para fortalecer la vigilancia microbiológica y de la resistencia antimicrobiana en sistemas de agua asociados a puntos de entrada internacionales, así como para el desarrollo de futuras investigaciones orientadas a esclarecer los mecanismos de persistencia, dispersión y resistencia de estos microorganismos en entornos aeroportuarios.
